# Construction and Validation of Subject-Specific Biventricular Finite-Element Models of Healthy and Failing Swine Hearts From High-Resolution DT-MRI

**DOI:** 10.3389/fphys.2018.00539

**Published:** 2018-05-29

**Authors:** Kevin L. Sack, Eric Aliotta, Daniel B. Ennis, Jenny S. Choy, Ghassan S. Kassab, Julius M. Guccione, Thomas Franz

**Affiliations:** ^1^Division of Biomedical Engineering, Department of Human Biology, University of Cape Town, Cape Town, South Africa; ^2^Department of Surgery, University of California, San Francisco, San Francisco, CA, United States; ^3^Department of Radiological Sciences, University of California, Los Angeles, Los Angeles, CA, United States; ^4^California Medical Innovations Institute, Inc., San Diego, CA, United States; ^5^Bioengineering Science Research Group, Engineering Sciences, Faculty of Engineering and the Environment, University of Southampton, Southampton, United Kingdom

**Keywords:** heart failure, subject-specific, finite element method, realistic simulation, ventricular function

## Abstract

Predictive computational modeling has revolutionized classical engineering disciplines and is in the process of transforming cardiovascular research. This is particularly relevant for investigating emergent therapies for heart failure, which remains a leading cause of death globally. The creation of subject-specific biventricular computational cardiac models has been a long-term endeavor within the biomedical engineering community. Using high resolution (0.3 × 0.3 × 0.8 mm) *ex vivo* data, we constructed a precise fully subject-specific biventricular finite-element model of healthy and failing swine hearts. Each model includes fully subject-specific geometries, myofiber architecture and, in the case of the failing heart, fibrotic tissue distribution. Passive and active material properties are prescribed using hyperelastic strain energy functions that define a nearly incompressible, orthotropic material capable of contractile function. These materials were calibrated using a sophisticated multistep approach to match orthotropic tri-axial shear data as well as subject-specific hemodynamic ventricular targets for pressure and volume to ensure realistic cardiac function. Each mechanically beating heart is coupled with a lumped-parameter representation of the circulatory system, allowing for a closed-loop definition of cardiovascular flow. The circulatory model incorporates unidirectional fluid exchanges driven by pressure gradients of the model, which in turn are driven by the mechanically beating heart. This creates a computationally meaningful representation of the dynamic beating of the heart coupled with the circulatory system. Each model was calibrated using subject-specific experimental data and compared with independent *in vivo* strain data obtained from echocardiography. Our methods produced highly detailed representations of swine hearts that function mechanically in a remarkably similar manner to the *in vivo* subject-specific strains on a global and regional comparison. The degree of subject-specificity included in the models represents a milestone for modeling efforts that captures realism of the whole heart. This study establishes a foundation for future computational studies that can apply these validated methods to advance cardiac mechanics research.

## 1. Introduction

For decades researchers have strived to create realistic computational models to represent the mechanical behavior of the heart (Sack et al., 2016a). This challenging endeavor faces difficulties in accounting for the complex geometry, fiber structure and material description of the heart. To further complicate modeling efforts, the circulatory system and the cyclical function of the heart need to be numerically reproduced as heart function is critically coupled to the circulatory system and cannot be modeled in isolation.

The finite element (FE) method is well suited to create computational models as it allows for partial differential equations to be solved over complex geometric domains, as is necessary to investigate the mechanical aspects of heart function, pathology and potential emergent therapies. This is critical as heart failure (HF) is the leading cause of death worldwide (Finegold et al., 2013). Even with optimal modern therapy, the annual mortality rate of patients with HF ranges from 31 to 45% (Chen et al., 2013; Desta et al., 2015), strongly motivating the need for new therapies, and methods that can accelerate their design and development. Modeling HF *in silico* allows the effect of the disease on heart function to be directly quantified (Bogen et al., 1980; Guccione et al., 2001; Kerckhoffs et al., 2007; Fomovsky et al., 2011; Wenk et al., 2011, 2012a,b) while simultaneously collecting critical information such as regional ventricular wall stress, an otherwise unobtainable metric thought to initiate pathological remodeling (Pfeffer and Braunwald, 1990; Sutton and Sharpe, 2000; Matiwala and Margulies, 2004).

Here, we propose a method to combine multiple sources of *in vivo* and *ex vivo* data to produce and validate highly realistic subject-specific FE models of the porcine heart. This process builds on our previously published research on cardiac modeling (Baillargeon et al., 2014, 2015; Sack et al., 2016b) by including subject-specific features into almost every aspect of the model to reduce the number of *ad hoc* modeling assumptions. By incorporating data from high resolution magnetic resonance imaging (MRI) and diffusion tensor magnetic resonance imaging (DT-MRI), we were able to create high-fidelity representations of the biventricular chambers, myofibers and infarcted scar-tissue distribution in the ischemic HF subject. These models include the full ventricular structure, the endocardial papillary structure and all four valve openings. Our modeling techniques simulate heart function by calibrating active and passive material properties of the heart to match measured *in vivo* functional outputs (i.e., volume and pressure measurements). To ensure realistic cardiac function, the mechanical model of the ventricles is coupled to a lumped-parameter circulatory model. This enables closed-loop volume exchange, the modeling of multiple cardiac cycles, and realistic cyclical pumping akin to the physiological beating heart. The models are validated by comparing predicted regional values of endocardial strain to *in vivo* measurements not used in model creation. This study introduces the first fully subject-specific cardiac models in healthy and failing states.

## 2. Methods

### 2.1. Experimental protocol

All animal experiments were performed in accordance with national and local ethical guidelines, including the Guide for the Care and Use of Laboratory Animals, the Public Health Service Policy on Humane Care and Use of Laboratory Animals, the Animal Welfare Act, and an approved California Medical Innovations Institute IACUC protocol regarding the use of animals in research.

Two porcine subjects were used in this study: one normal and one with HF. The description of these animals and the creation of HF has been detailed previously (Choy et al., 2018). The ischemia resulted in decline of the animal's ejection fraction (EF) from 56% at the time of coronary artery occlusion to 32% when the animal was sacrificed, 16 weeks later. Measurements of *in vivo* left ventricular pressure and volume for each subject were recorded at the time of sacrifice (the incorporation of this data is discussed in section 2.5). Excised hearts were arrested in diastole with a saturated solution of potassium chloride and were fixed with buffered formalin (Carson-Millonig formulation).

### 2.2. *Ex Vivo* imaging

After fixation, the ventricular cavities were filled with a silicone rubber compound (Polyvinylsiloxane, Microsonic Inc., Ambridge, PA) in order to maintain the geometry during imaging. The hearts were then placed in a plastic cylindrical container filled with a susceptibility-matched fluid (Fomblin, Solvay Solexis, West Deptford, NJ) and held in place using open-cell foam. Anatomical MRI was then performed (Magnetom Prisma 3T, Siemens, Erlangen, Germany) with the following parameters: T1-weighted imaging using a 3D Fast Low Angle SHot (FLASH) sequence (0.3 × 0.3 × 0.8 mm spatial resolution, echo time (TE)/repetition time (TR) = 3.15/12 ms, scan time: 1.5 h); and T2-weighted imaging using a 2D multi-slice Turbo Spin Echo (TSE) sequence (0.3 × 0.3 × 0.8 mm spatial resolution, TE/TR = 94/15,460 ms, scan time: 2 h).

DT-MRI was performed using a readout-segmented diffusion-weighted spin-echo sequence (Porter and Heidemann, 2009) with b-value = 1,000 s/mm^2^ along 30 directions and one b-value = 0 s/mm^2^ reference, TE/TR = 62/18,100 ms and 1.0 × 1.0 × 1.0 mm spatial resolution with 4–6 signal averages to improve signal-to-noise ratio (scan time: 8–12 h). Diffusion tensors were reconstructed from the diffusion-weighted images using linear regression and custom MATLAB (The MathWorks, Inc., Natick, Massachusetts, United States) routines.

### 2.3. Geometric segmentation and reconstruction

*Ex vivo* MRI data sets were imported and processed in Simpleware ScanIP (Synopsys, Mountain View, USA). Detailed geometric segmentations of the biventricular structure were created along with segmentations of infarcted tissue in the HF subject. Segmentation relied on a combination of well-established techniques including region growing, level-set thresholding, and morphological smoothing (Vadakkumpadan et al., 2010; Setarehdan and Singh, 2012). Manual intervention was used only if needed to eliminate spurious features.

**Figure 1:**
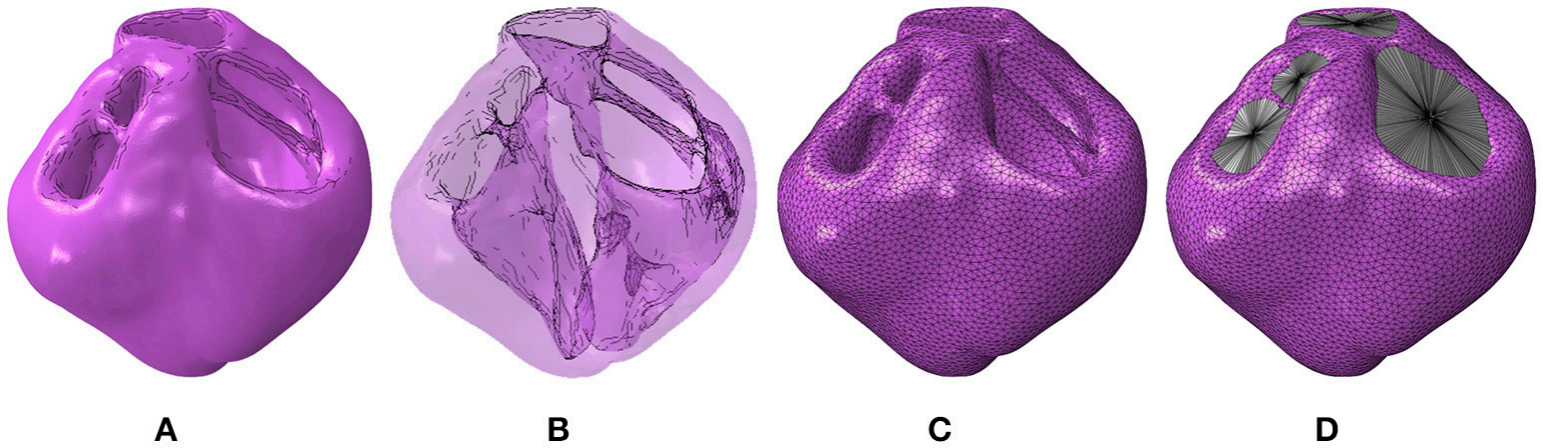
**(A)** Geometric segmentation. **(B)** Transparent geometries revealing the cavity morphology. **(C)** The mesh corresponding to the segmentation. **(D)** The mesh with the cavity structures enclosed using surface elements.

The full ventricular structure including all four valve openings of the heart was reconstructed from the T2-weighted MRI data sets. The segmented geometry and the cavity morphology are shown in Figures 1A,B, respectively. The segmented geometry was meshed with quadratic tetrahedral elements using Simpleware's built in FE meshing suite as shown in Figure 1C and the resultant mesh with the cavities enclosed is shown in Figure 1D. These meshes were imported into the Abaqus software environment (version 6.14, Dasssult Systèmes, Providence, RI, USA), which was chosen as the FE solver for this research. Since these geometries are constructed from *ex vivo* imaging, they provide the geometry in an unloaded state.

#### 2.3.1. Ventricular chambers

To determine the ventricular cavity volumes, these chambers were enclosed by constructing two-dimensional (2D) triangular surface elements at each valve opening that were adjoined to a center node Figure 1D. The degrees of freedom of these center nodes, designated as “slave nodes,” were coupled to the average motion of the surrounding nodes on the ventricular structure used to construct these surface elements. These surface elements do not contribute to the stiffness of the valve openings.

### 2.4. 3D subject-specific myofiber orientations

DT-MRI provides diffusion tensors for each voxel that were decomposed into eigenvalues and corresponding eigenvectors. Primary eigenvectors associated with the largest eigenvalue were identified as the orientation of the myofiber (Scollan et al., 1998; Kung et al., 2011). For practical purposes of computational modeling, a fully continuous 3D field representation of the local material coordinates, derived from diffusion tensors, is needed. This was achieved using a linear invariant interpolation method (Gahm et al., 2012) whereby the diffusion tensor is decomposed into invariants and orientations, which are each interpolated in turn and reconstructed into an interpolated tensor at the point of interest **x** ∈ ℝ^3^.

To ensure only voxels containing cardiac tissue (and not fat, air bubbles or voids) were included in the interpolation, the requirements that eigenvalues of each voxel be strictly positive, and that the fractional anisotropy (FA), an invariant of diffusion tensors commonly used for tissue thresholding (FA > 0.12), were imposed prior to analysis. This value for FA was found experimentally to be the lowest that would fully threshold out non-fibrous tissue and was reasonably different from values of FA for cardiac tissue (Helm et al., 2005; Kung et al., 2011). The inclination angle α_h_, defined as the angle between the myofiber projected onto the longitudinal-circumferential tangent plane and the circumferential unit vector (Bovendeerd et al., 1992; Scollan et al., 1998; Toussaint et al., 2013), was quantified for each voxel. Results are presented regionally for the left ventricle (LV), partitioned into the 17 regions following the American Heart Association (AHA) guidelines (Cerqueira et al., 2002). This is a commonly reported quantification of myofiber orientation, which allows us to compare our results with literature findings as a source of validation.

### 2.5. Incorporating *in vivo* measurements

For the HF pig, *in vivo* pressure and volume measurements were set as target values in the model calibration. For the normal pig, an *in vivo* volume measurement, and pressure derived from the healthy baseline of a larger *in vivo* data set (*n* = 5) (Choy et al., 2018), were similarly used. The complete *in vivo* measurements used for this study are presented in Supplementary Table S1. Measurements of *in vivo* strains were also recorded but deliberately excluded from the calibration process to serve as an independent metric to validate the model.

### 2.6. Constitutive model

#### 2.6.1. Passive material description and parameter estimation

The passive material response for myocardium follows the structurally motivated constitutive model for anisotropic hyperelastic myocardium introduced by Holzapfel and Ogden (Holzapfel and Ogden, 2009). Descriptions of material parameters are provided in Supplementary Table S2. A modification in the isochoric part of the strain energy density Ψ_*iso*_, was introduced, allowing for the description of homogenized, pathological tissue:

(1) $$\begin{array}{l} \Psi_{iso}\;=\;\frac{\bar{a}}{2b}e^{b(I_{1}-3)}+\;\sum_{i=f,s}^{\;}\frac{{\bar{a}}_{i}}{2b_{i}}\left\{e^{b_{i}{\left(I_{4i}-1\right)}^{2}}-1\right\}\\ \;+\;\frac{{\bar{a}}_{fs}}{2b_{fs}}\left\{e^{b_{fs}{\left(I_{8fs}\right)}^{2}}-1\right\}, \end{array}$$

(2) $$\Psi_{vol}\;=\;\frac{1}{D}\left(\frac{J^{2}-1}{2}-\ln(J)\right)$$

where the new parameters $\bar{a}$, ${\bar{a}}_{i}$ and ${\bar{a}}_{fs}$ govern the homogenization of healthy and pathological tissue using a scalar parameter *h* representing the volume fraction of tissue health. For example ${\bar{a}}_{i}$ is defined in the following manner:

(3) $${\bar{a}}_{i}=\;a_{i}\left[h+\left(1-h\right)p\right].$$

Here, *h* bound by [0, 1], governs the health of the material point and *p* scales the passive response according to pathology. The parameters $\bar{a}$ and ${\bar{a}}_{fs}$ are defined similarly using *h, p*, *a* and *a*_*fs*_. Note that the following holds:

(4) $$h\;=1\overset{yields}{\to }{\bar{a}}_{i}=a_{i}$$

(5) $$h\;=0\;\overset{yields}{\to }{\bar{a}}_{i}=a_{i}p$$

${\bar{a}}_{i}$ transitions linearly between these values for different values of *h* bound by [0, 1].

**Figure 2:**
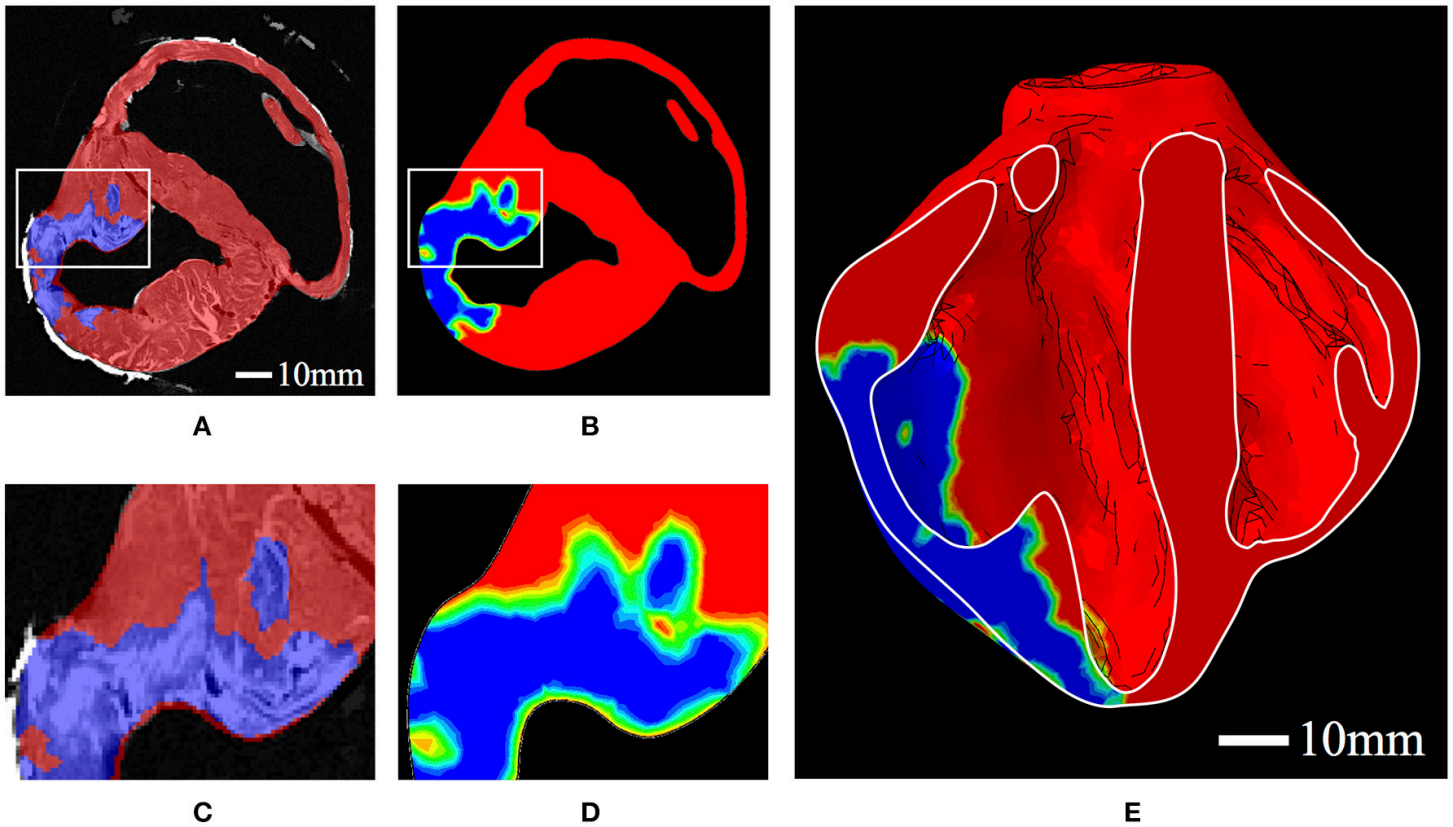
**(A)** Binary segmentation of infarcted tissue (blue) and healthy tissue (red) on a short axis MRI of the porcine subject with HF. **(B)** A short-axis slice of the FE model displaying the interpolated *h* field with 0 represented as blue and 1 represented as red. Colors in between blue and red represent the “border zone.” **(C)** Zoomed-in section corresponding to the box in **(A)**. **(D)** Zoomed-in section corresponding to the box in **(B)**. **(E)** A long-axis cut plane of the FE model displaying the interpolated *h* field throughout the bisected geometry.

The values of *h* were determined from *ex vivo* imaging and processed as a regionally varying field, continuous over the domain. This was achieved by interpolating a binary (i.e., infarcted or healthy tissue) segmentation of the high-resolution *ex vivo* data onto regularly spaced nodal points of the biventricular FE mesh, whereby the elemental interpolants populate the domain in a continuous fashion. This allows for regionally detailed descriptions of infarcted tissue and border zone material to be incorporated into the model in a continuous and physiologically reasonable manner (Figure 2). Following the above equations, passive material stiffness is determined by the “health” of the material point, *h*; the pathological scaling of infarcted material, *p*, and the material parameters *a*_*i*_ and *b*_*i*_, which govern the linear and exponential response of the cardiac tissue in different modes of deformation.

*h* is determined *a priori* from the mapping of the segmented infarcted tissue. Without detailed experimental data, we have to rely on literature values to determine the pathological scaling of the material, *p*. Even though multiple studies have investigated the quantification of infarct mechanics, there is no clear consensus on infarct stiffness readily available. Holmes, Borg (Holmes et al., 2005) presents an excellent account of changes in infarct stiffness. The infarct at the remodeling phase (i.e., scar tissue) is relevant to our study, and has been quantified as 2–10 times (Connelly et al., 1985; Gupta et al., 1994; McGarvey et al., 2015; Mojsejenko et al., 2015) as stiff as remote non-infarcted tissue. Particularly relevant is a recent study performed by McGarvey, Mojsejenko (McGarvey et al., 2015) which allows us to narrow this range of infarct stiffness. In that study, the authors quantify the *in vivo* stiffness of infarct and remote tissue of porcine subjects using FE methods and *in vivo* imaging techniques. As they have a similar model of MI and report values for late-stage infarcts (i.e., 12 weeks) their results are most applicable to our study. Using the ratio of their results for infarcted and remote tissue, we determine a value for *p* to be 4.56 which we apply throughout our models.

The remaining material parameters, *a*_*i*_ and *b*_*i*_, were found through optimization techniques relying on two stages of determination. Initial values for *a*_*i*_ and *b*_*i*_ were determined from the calibration of normal myocardium specimen samples to experimental tri-axial shear data (Sommer et al., 2015). This is essential to capture the fully orthotropic behavior of cardiac tissue. Calibration was performed using Abaqus as the forward solver, whereby *in silico* cubes of myocardium with edge lengths of 4 mm (i.e. dimensions matching those of the study of interest) were meshed into a uniform 27 linear hex-element mesh. As with Sommer et al. (2015), we assumed an orthonormal coordinate system aligned with the cube dimensions corresponding to the mean fiber, sheet, and sheet-normal directions. Shearing was executed by specifying the translational displacement of a specified cube face, while enforcing zero displacement boundary conditions on the opposite cube face. The optimization was performed in MATLAB using a nonlinear least-square optimization routine with the trust-region-reflective algorithm option.

Here, the minimization between FE model stress σ and experimental values $\bar{\sigma }$ can be explicitly defined through the minimization of an objective function φ_1_ by

(6) $$\text{min }\varphi_{1}(v_{1})=\sum_{i}^{\;}\sum_{j}^{\;}{\left(\sigma_{j}^{i}-{\bar{\sigma }}_{j}^{i}\right)}^{2},$$

where *i* = {*fs, fn, sf, sn, nf, ns*} are the six combinations of shear modes, the vector of material parameters is given by **v**_**1**_ = {*a, b, a*_*f*_, *b*_*f*_, *a*_*s*_, *b*_*s*_, *a*_*fs*_, *b*_*fs*_} and the index *j* spans the data points in the shear vector for shear test *i*. The resulting material parameters from shear calibration were identified only once and these formed as the starting set of material parameters for the next stage of calibration, which scales these values to match subject specific left ventricular function.

To adjust the material for each subject, these initial values are scaled consistently to match the “Klotz curve” (Klotz et al., 2006) generated for the diastolic pressure-volume (PV) relation of each subject's LV. Both linear (*a*_*i*_) and exponential (*b*_*i*_) terms were subject to uniform scaling by parameters *A* and *B*, a scalar and an exponential multiplier, respectively. These values were found by minimizing the error between the *in silico* diastolic PV course of each subject to the analytical Klotz curve, starting from the unloaded LV volume V_0_ until the end-diastolic volume (EDV) was reached at the specified end-diastolic pressure (EDP) value given in Supplementary Table S1. The error between the model and predicted *in vivo* pressure-volume relationship was minimized using the same nonlinear least-square optimization routine used in the shear calibration. For the passive filling calibration, we defined our objective function φ_2_ as the difference in pressure values along the pressure volume curve combined with a single measure of EDV, which we found to yield close fits to the PV curve and ensure EDV was met.

(7) $$\min\varphi_{2}(v_{2})=\sum_{j}^{N}{\left(P_{j}-{\bar{P}}_{j}\;\right)}^{2}+{\left(EDV-\bar{EDV}\right)}^{2}\;,$$

where the vector of material parameters is given by **v**_**2**_ = {*A, B*}, *N* refers to the total number of data points along the pressure volume curve and values from experimental data are given with the “overbar” notation. To be thorough, the enforcement of incompressibility was investigated by perturbing the parameter *D* in Eq. (2). We found that at extreme values, i.e., *D* < 0.02 MPa and *D* > 20 MPa, non–physiological deformation was introduced. Within this range (0.02 < *D* < 20 MPa), the effect of incompressibility was minor on material parameter estimation. We chose to set *D* = 0.2, which we found sufficient to enforce incompressibility (99.8% volume retained over passive filling) and avoid problematic deformations. This value produced a Bulk modulus roughly 1000 times larger than the largest linear terms (*a*_*i*_) – a guideline also used by Göktepe et al. (2011).

To ensure realistic loading of the LV cavity one needs to consider the trans-septal pressure originating from RV filling. To capture this, the RV cavity of the normal subject was also inflated to 4 mmHg for RV EDP during passive filling calibration. This amount was determined from literature values of healthy subjects (Quinn et al., 2006; Mann et al., 2015). As the HF subject had an LV EDP roughly double the LV EDP of the normal subject, we also doubled the RV passive pressure to maintain proportionality.

#### 2.6.2. Active material description and parameter estimation

The description of our time-varying elastance model of active force development (Walker et al., 2005) was also modified to include this description of tissue health:

(8) $$T_{a}(t,l)=T_{max}\frac{Ca_{0}^{2}}{Ca_{0}^{2}+ECa_{50}^{2}(l)}\;\frac{\left[1-\cos(\omega (mod(t),l))\right]}{2}h,$$

where *T*_*max*_, the maximum allowable active tension, is multiplied with a term governing the calcium concentration, and a term governing the timing of contraction (both terms depend on sarcomere length *l*). The timing of contractile function is linked to the heart rate and timing of the cardiac cycle, which is enforced through a modulus function acting on the time variable, *t*. Finally, the entire expression is multiplied by *h*, to ensure tissue contractility is directly proportional to tissue health. This ensures contractile force is zero at the material point of fully infarcted tissue. Further detail of the active tension law is provided in the Supplementary Material.

The total fiber stress, σ_***f***_, is equal to the passive stress, σ_***pf***_, combined with the active tension in the fiber direction given by:

(9) $$\sigma_{f}=\sigma_{pf}+T_{a\;}{\text{e}}_{\text{f}}⊗{\text{e}}_{\text{f}}$$

Biaxial investigations on actively contracting rabbit myocardium revealed significant stress development in the cross-fiber direction that could not be completely attributed to fiber dispersion or deformation effects (Lin and Yin, 1998). This has motivated computational efforts to consider a proportion of the active stress developed in the fiber direction to be transferred onto the stress in the sheet direction by a scalar *n*_*s*_ ∈ (0, 1), such that:

(10) $$\sigma_{s}=\sigma_{ps}+n_{s}\;T_{a\;}e_{s}⊗e_{s}$$

Using the same nonlinear least-square optimization routine in the passive regime, *T*_max_ and *n*_*s*_ were subjected to optimization to ensure the correct stroke volume (SV = EDV-ESV) for each subject was achieved. Additionally, to ensure physiological deformation during contraction (and unique values of parameter estimation), left ventricular long-axis shortening (LVLS) was included in the description of error for the optimization routine. Typical values for LVLS are between 15 and 20% for humans (Dumesnil et al., 1979; Carreras et al., 2012), so this was set as a low weighted target in the minimization routine to ensure an LVLS > 0% in our *in silico* porcine model was achieved. This in turn ensures that the optimization routine does not converge on a parameter set that produces ventricular elongation and/or wall thinning. This is defined explicitly in the objective function φ_3_ below:

(11) $$\min\varphi_{3}\left(v_{3}\right)={\left(SV-\bar{SV}\right)}^{2}+0.2\;{\left(LVLS-\bar{LVLS}\right)}^{2}$$

where the vector of active material parameters is given by **v**_**3**_ = {*T*_max_, *n*_*s*_} and target values SV and LVLS are given with the “overbar” notation.

### 2.7. Circulatory system

**Figure 3:**
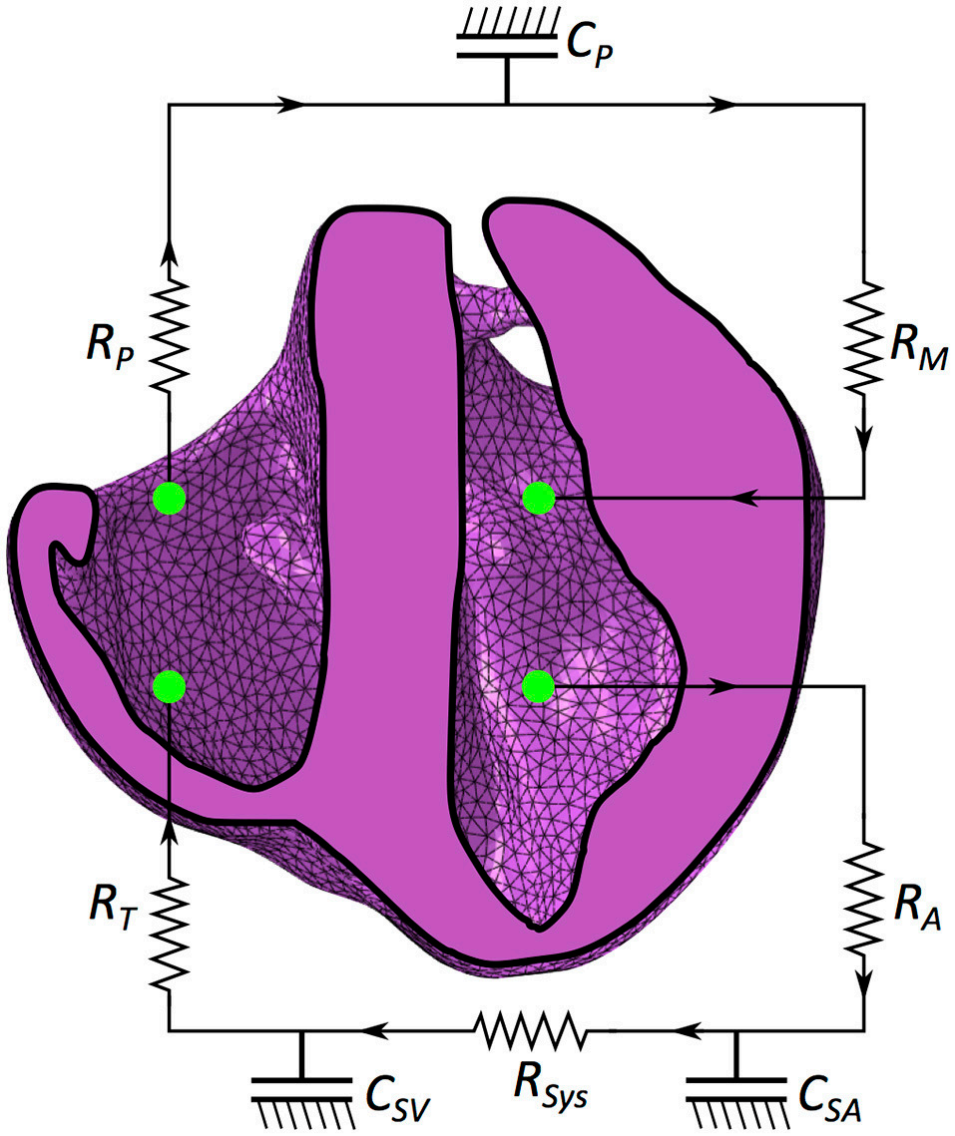
Schematic of the mechanical model coupled with the circulatory system. *R*_*M*_ is mitral valve resistance, *R*_*A*_ is aortic valve resistance, *C*_*SA*_ is systemic arterial compliance, *R*_*SYS*_ is systemic arterial resistance, *C*_*SV*_ is venous compliance, *R*_*T*_ is tricuspid resistance, *R*_*P*_ is pulmonary valve resistance and *C*_*P*_ is pulmonary system compliance.

We introduced a closed loop circulatory model adapted from simple lumped parameter representations (Hoppensteadt and Peskin, 1992; Pilla et al., 2009) of different compartments in the cardiovascular system. The ventricular chambers are defined as fluid-filled cavities fully enclosed by the combined meshed faces of the tetrahedral elements on the cavity surface and the surface elements described in section 2.3.1 that close off the chamber. The coupling of the lumped circulatory system and mechanical function was performed in Abaqus. Details on the numerical underpinnings for this are provided in the Abaqus Theory Guide (2014); here, we provide a brief overview concerning the relation of pressure, volume, compliance, resistance and fluid exchange within a lumped system. The volume *V*_*i*_ and pressure *P*_*i*_ inside a fluid cavity chamber *i* are related in the following manner:

(12) $$V_{i}\left(t\right)=V_{i}\left(0\right)+\kappa_{i}P_{i}\left(t\right)$$

where κ_*i*_ is the compliance (inverse of stiffness) of the vasculature. For the LV and RV, the compliance is highly nonlinear, depending on both the strain state of the material and time (outlined in sections 2.6.1 and 2.6.2). Following Eq. (12), we define additional dimensionless compliance vasculature representing key components of the circulatory system. As the FE ventricular model (and not the circulatory circuit) is the central focus of this research, we sought to model a working circulatory system with as few components and assumptions as possible. This resulted in a circulatory system with three compliance vessels representing the systemic arteries (*SA*), systemic veins (*SV*) and the pulmonary circuit (*P*). The FE model of the heart is connected to this lumped representation as illustrated in Figure 3.

Unidirectional fluid exchanges governing the flow between compliance vessels are driven by the pressure gradients between these chambers as defined by:

(13) $$Q\left(t\right)=\frac{dV}{dt}=\frac{1}{R}\frac{dP}{dt}$$

In each simulated cardiac cycle, the ventricles contract, increasing the pressure in their chambers until it exceeds the pressure in the connected outflow chamber, driving the flow of blood in the circuit and simulating the physiological circulatory system. Since the total amount of volume in the circulatory system is constant, we have the following:

(14) $$\frac{d}{dt}V_{TOT}=\frac{d}{dt}\sum_{i}^{\;}V_{i}\left(t\right)=0.$$

This facilitates a stable limit cycle when simulations occur over multiple cardiac cycles.

Resistance and compliance values were based on literature values (Santamore and Burkhoff, 1991; Hoppensteadt and Peskin, 1992; Watanabe et al., 2004; Pilla et al., 2009) and adapted to ensure realistic flow. The values for these parameters are presented in Supplementary Table S3 along with literature values for comparison.

Flows from the pulmonary circuit into the LV and from the systemic circuit into the RV are set to zero during the contractile stages of heart function (i.e., during isovolumetric contraction, ejection, and isovolumetric relation). Outflow from the LV and RV is always permissible but only occurs when the pressures in these cavities exceed the pressures in the chambers they are ejecting into. Combining these restraints with the imposed unidirectional flow of the circuit is sufficient to produce realistic fluid exchanges analogous to the physiological circulatory system.

### 2.8. Boundary conditions

The physiological heart does not experience rigid constraints on its motion. Boundary conditions are needed in computational models, however, to prevent rigid body motion and ensure the problem is mathematically well posed. To accomplish this without placing overly restrictive constraints on the model motion, we exploited the coupled degrees of freedom introduced by enclosing the ventricular cavities (section 2.3.1). The slave node of the truncated pulmonary trunk was fixed in all directions; this indirectly enforces a weighted average restraint on the nodes it is coupled with; i.e., the valve ring, such that the average deformation is zero. The valve is still able to expand and contract during the heart cycle, but this motion is “centered” on a fixed point in space.

### 2.9. Initial conditions

In order to initiate the cardiac cycle, each compliance vessel was loaded with fluid until it experienced the physiological pressure at ED. Where possible, pressure catheterization readings from *in vivo* subject-specific measurements were used. The set of initial conditions used in this study is given in Supplementary Table S4.

### 2.10. The *in silico* cardiac cycle

The initial conditions outlined above are sufficient to initiate the dynamic beating of the computational heart model. From the initial conditions (i.e., ED), contraction is initiated, which increases the pressure in the ventricular chambers. These rise until LV and RV pressures exceed the pressures in *C*_*SA*_ and *C*_*p*_, respectively. At this stage, fluid exchanges occur and the ventricles empty while the pressures and volumes in *C*_*SA*_ and *C*_*p*_ increase. Once LV and RV pressures drop below the pressures in *C*_*SA*_ and *C*_*p*_ respectively, the fluid exchanges stop, and the LV and RV pressures decrease with the decline in active tension. The entire duration of these “active contraction” processes is 480 ms, which compares well with literature values for the timing of isovolumetric contraction 66–90 ms (Sengupta et al., 2006), ejection 270–347 ms (Beyar and Sideman, 1984a; Sengupta et al., 2006) and isovolumetric relaxation 64–93 ms (Hanrath et al., 1980; Sengupta et al., 2006), respectively.

At the end of isovolumetric relaxation, all components in the circulatory system behave purely passively. Pressure differences between the compliance chambers in *C*_*SV*_ and *C*_*p*_ and the RV and LV, respectively, drive the passive filling of the ventricular chambers. The majority of the volume transferred in the passive filling stage occurs in the early portion of the step when the pressure difference is largest. Passive filling occurs in 300 ms, which is sufficient time for the cavities to inflate to the ED state and results in a heart rate of 77 bpm. Multiple steps of active contraction and passive filling can be simulated in a continuous sequence until convergent behavior over the cardiac cycle is reached.

### 2.11. Damping

Mass proportional Rayleigh damping (i.e., a viscous term introduced in the FE system of equations proportional to the mass matrix; Hughes, 2000) is introduced to dampen unrealistic oscillatory behavior of the low frequency modes. Physiologically, these would be eliminated by the surrounding soft connective tissue in the chest cavity. Similarly, isotropic time-dependent linear viscoelasticity is defined as part of the material constitutive behavior to damp out the high frequency response during active contraction. Whereas cardiac tissue is generally known to exhibit viscoelastic behavior, suitable experimental data on porcine cardiac viscoelasticity were not available. Hence, the model incorporates a small amount of viscoelasticity to eliminate unrealistic transient behavior, which is achieved using a Prony series formulation (Dill, 2006) within the Abaqus material definition (Abaqus Theory Guide, 2014).

### 2.12. Model validation

*In vivo* strain echo data (TomTec 4D LV-Function, Version 4.6, Build 4.6.3.9, Unterschleißheim, Germany) of the endocardial surface was collected and excluded from calibration to serve as an independent data source to perform model validation. These *in vivo* strains are calculated by partitioning the endocardial surface into 16 segments, and measuring local deformation in longitudinal and circumferential directions (Pedrizzetti et al., 2014). An illustration of this 16-segment partition is shown in Supplementary Figures S1A,B.

These *in vivo* strain measurements reference ED as the initial configuration, and as such, provide relative change in length through a single cardiac cycle compared to the ED state. For purposes of validation, we select ES as the primary point of comparison for measuring heart deformation.

To provide comparable strain measures from our FE model, the endocardial nodes were partitioned into the same 16 segment division as the TomTec strain data. This resulted in 12 quadrilateral segments for the LV trunk and four triangular segments for the apical region. Control nodes placed at the corners and midpoints of the regions were identified. Similarly to the speckle-tracking imaging technique used to determine strain in the *in vivo* case, the nodal deformations of these control points were extracted at different time points in the cardiac cycle. By fitting cubic splines through these control points, longitudinal and circumferential measurements are created. The change in longitudinal and circumferential spline length provides a consistent strain measurement (i.e., engineering strain) analogous to the strain measurements provided by the *in vivo* TomTec strain measurements. The quadrilateral and triangular surfaces, the control nodes, and the fitted cubic splines are illustrated in Supplementary Figures S1C,D.

## 3. Results

### 3.1. Geometric segmentation

**Figure 4:**
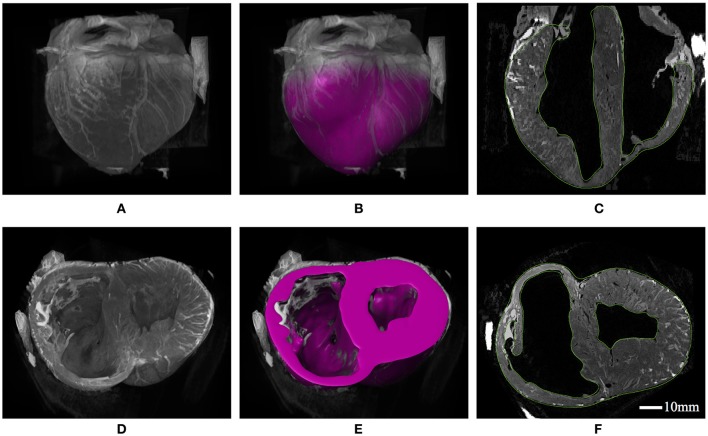
**(A)** 3D constructed visualizations of the full ventricular structure from MRI data. **(B)** Geometric segmentation of the full ventricular structure superimposed over the same background MRI data as **(A)**. **(C)** Long-axis cut plane of the MRI data superimposed with contours (green lines) for the full ventricular segmentation. **(D)** 3D constructed visualizations of the truncated ventricular structure from MRI data, revealing the endocardial ventricular structure. **(E)** Geometric segmentation of truncated ventricular structure superimposed over the same background MRI data in **(D)**. **(F)** Short-axis cut plane of the MRI data superimposed with contours (green lines) for the ventricular segmentation.

A visualization of the 3D heart structure from T2-weighted MRI data is given in Figure 4, along with our ventricular segmentation. Structural details such as wall thickness and trabecular morphology extracted from the MRI data conform to features reported for the porcine heart (Crick et al., 1998). Segmentations of the ventricles were meshed with roughly 85,000 quadratic tetrahedral elements–a refinement determined from a mesh convergence analysis. This analysis identified converged model behavior (i.e., stress, strain, and cavity expansion) over the entire cardiac cycle for mesh resolutions greater than approximately 50,000 elements.

### 3.2. Subject-specific myofiber orientations

**Figure 5:**
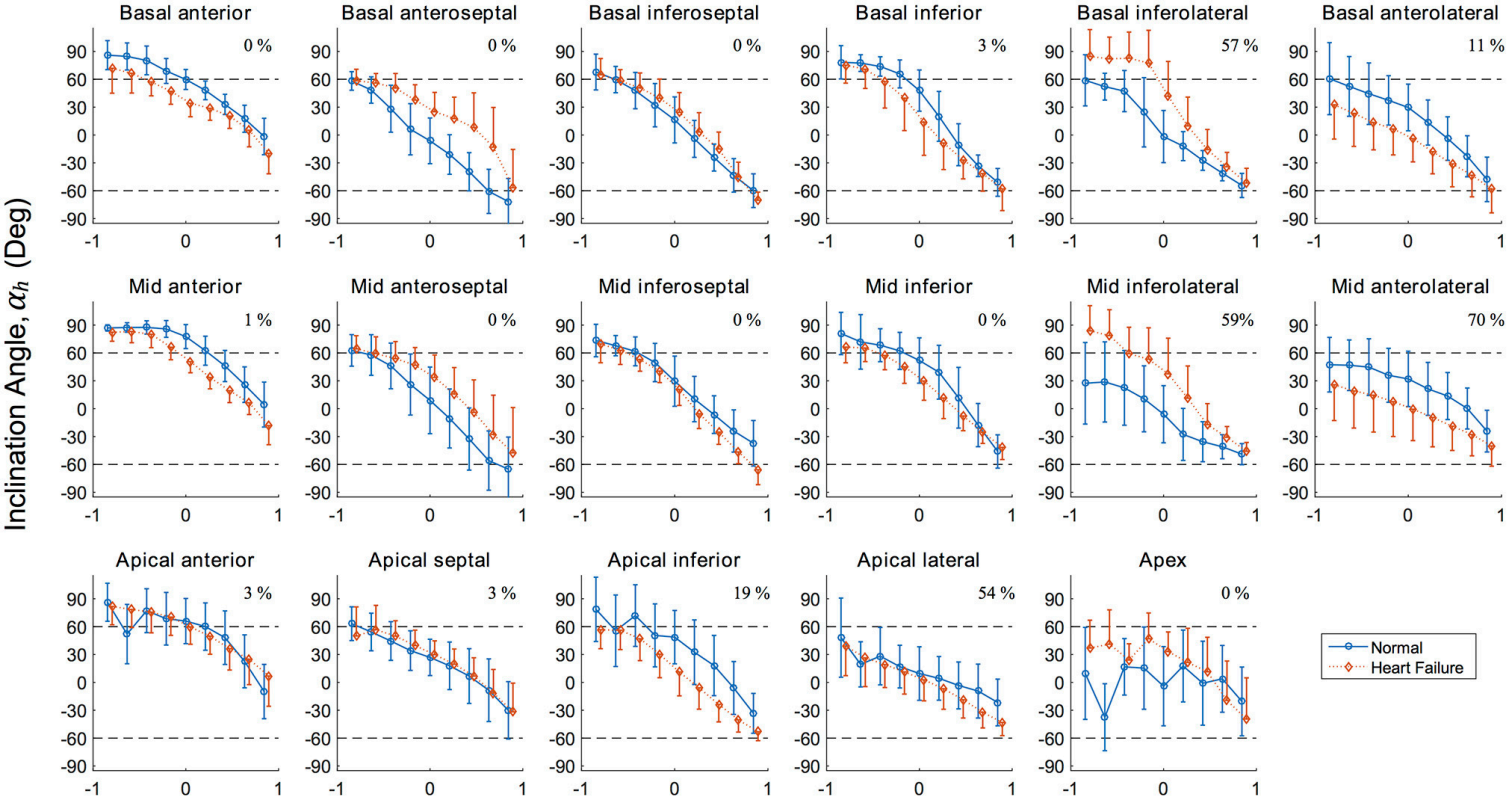
Inclination angles α_h_ in the 17 left ventricle regions (Cerqueira et al., 2002) showing the distribution along the radial depth for the normal (healthy) and diseased (heart failure) subjects. Normalized radial coordinates were used to indicated the endocardium (−1), mid wall (0) and epicardium positions (+1). Dashed lines corresponding to +60° and −60° are plotted for ease of comparison and because a significant number of studies use these bounds when prescribing α_h_ in LV computational models. Percentage given in top right corner of each panel corresponds to the degree of infarcted tissue in the AHA region for the heart failure model, calculated as 1–mean(*h*).

Results for the inclinations angles α_h_ for both porcine LVs are presented graphically in Figure 5 for each AHA segment. This regional presentation of α_h_, partitioned by AHA region and further subdivided by transverse wall depth, presents in general with narrow distributions (i.e., small variance). Inclination angles in the normal subject, excluding the apex, vary from 66.5 ± 16.6° on the endocardium to −37.4 ± 22.4° on the epicardium in a predominantly linear fashion. Similarly, inclination angles in the HF subject vary from 63.0 ± 18.3° on the endocardium to −43.4 ± 19.8° on the epicardium.

**Figure 6:**
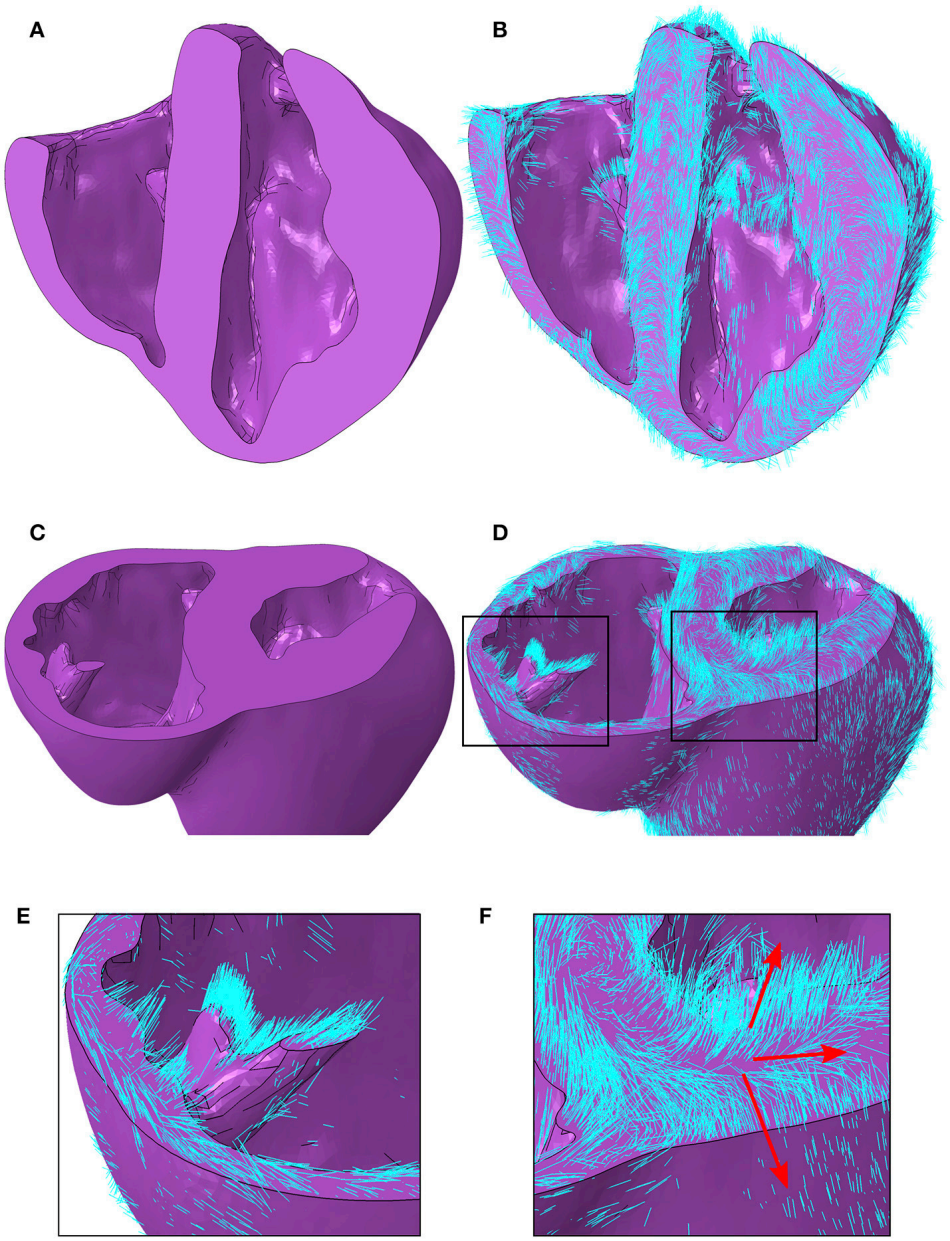
**(A)** Porcine geometry bisected longitudinally to reveal the endocardial surfaces. **(B)** Myofiber orientations plotted in cyan lines for the same geometry revealed in **(A)**. **(C)** Porcine geometry cut along a short axis to reveal cut papillary structures in the RV and the short-axis plane in the LV. **(D)** Myofiber orientations plotted in cyan lines for the same geometry revealed in **(C)**. **(E)** Zoomed-in image of the cut RV papillary structure with fibers from **(D)**. **(F)** Zoomed-in image of the LV short-axis plane from **(D)**. Red arrows aligned with the local myofiber orientation are added for regions in the epicardial, mid wall and endocardial regions. LV, left ventricle; RV, right ventricle.

A local orthonormal coordinate system aligned with the myofiber, sheet plane, and sheet normal directions are interpolated to the centroid of each element in the FE mesh. Images revealing the geometry with and without myofiber orientations are presented in Figure 6. In addition to the characteristics presented quantitatively above, other qualitative features are as follows. Firstly, myofiber orientations are predominantly tangential with geometric surfaces. This can be seen in Figures 6B,D, by the abundance of myofibers protruding through cut surfaces relative to those seen protruding through natural physiological surfaces. Secondly, myofibers are closely aligned with papillary structure morphology, as can be seen in Figures 6C–E. Finally, the distribution in inclination angle in the LV, varying from positive on the endocardium through to negative on the epicardium, is easily identified from the global myofiber arrangement as shown in Figure 6F.

### 3.3. Material parameter estimation

The calibrated material parameters fit the human shear data (Sommer et al., 2015) with an *R*^2^ = 0.997. This excellent fit is illustrated in Supplementary Figure S2 wherein model response (solid lines) is plotted against experimental data (circles). The corresponding material parameters that produced this response are a = 1.05 kPa, b = 7.542, a_f_ = 3.465(kPa), b_f_ = 14.472, a_s_ = 0.481(kPa), b_s_ = 12.548, a_fs_ = 0.283(kPa), and b_fs_ = 3.088.

The subject-specific calibration resulted in suitable values for the passive material scaling parameters *A* and *B*, and active material parameters *T*_*MAX*_ and *n*_*s*_. These values are presented in Table 1 along with the initial unloaded LV cavity volumes V_0_, as these are fundamental to the resulting material parameters.

**Table 1:** Initial volumes and calibrated material parameters for the normal and heart failure subjects.

**Pig**	**V_0_**	**A**	**B**	***T*_*MAX*_**	***n*_*s*_**
Normal	17.5	0.16	0.73	118.0	0.07
Heart failure	47.1	1.69	0.87	140.6	0.14

**Figure 7:**
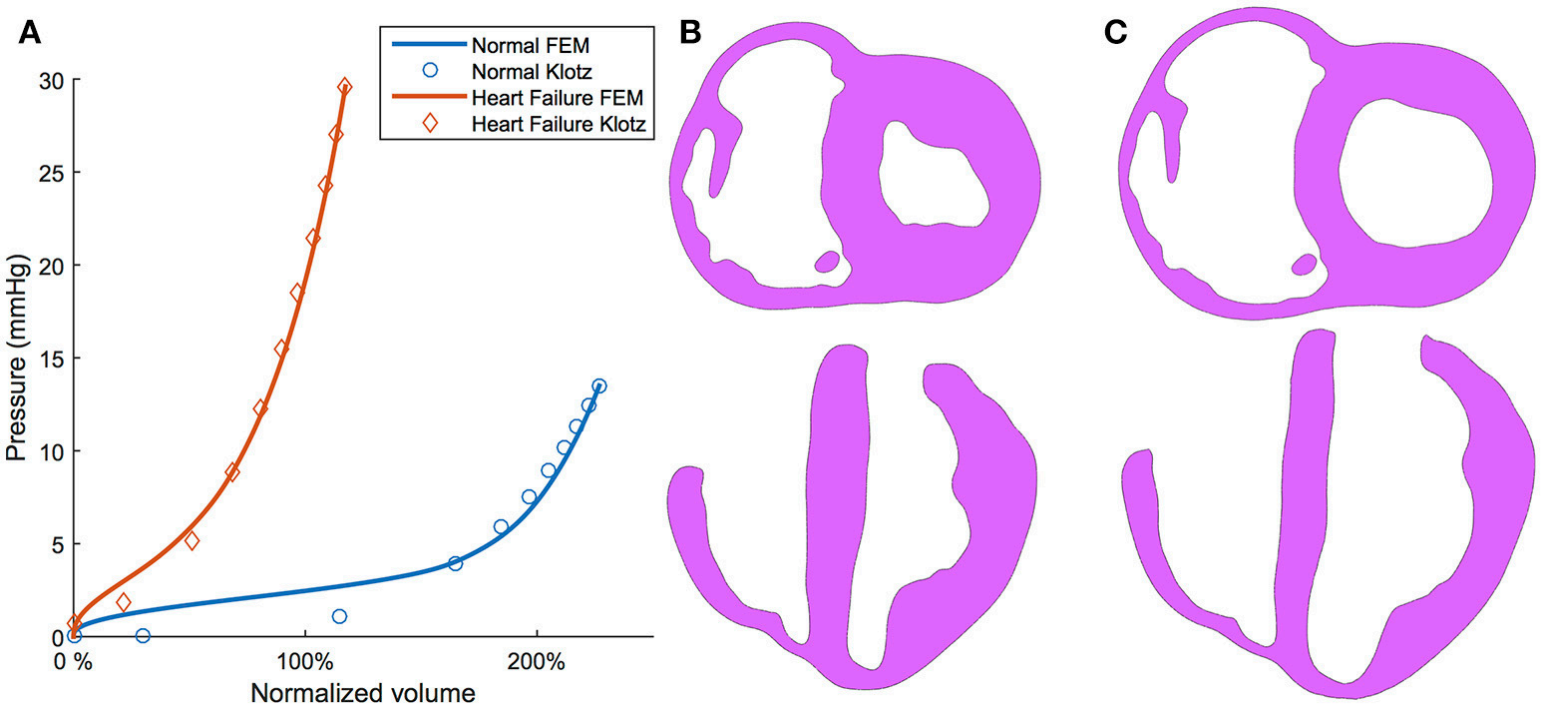
**(A)** Klotz curve (markers) and model response (solid lines) for the normal (healthy) and diseased (heart failure) subjects after calibration whereby the volume is nominalized to V_0_. **(B)** Long-axis and short-axis views of the ventricular structure at the unloaded configuration. **(C)** Long-axis and short-axis views of the ventricular structure at the end-diastole configuration.

To illustrate the efficacy of the optimization routine, the passive filling curve resulting from optimizing is given in Figure 7A. These passive filling curves fit the analytical Klotz curves with an *R*^2^ = 0.967 for the normal subject and an *R*^2^ = 0.995 for the HF subject. The final calibrated EDV also matches closely with target values; i.e., 57.3 vs. 57.8 ml (normal) and 102.4 vs. 103.0 ml (HF). Sample long- and short-axis profiles of the ventricular structure in the unloaded (i.e., initial) and the ED configuration are presented in Figures 7B,C for the normal subject.

### 3.4. Coupled mechanical and circulatory model

**Figure 8:**
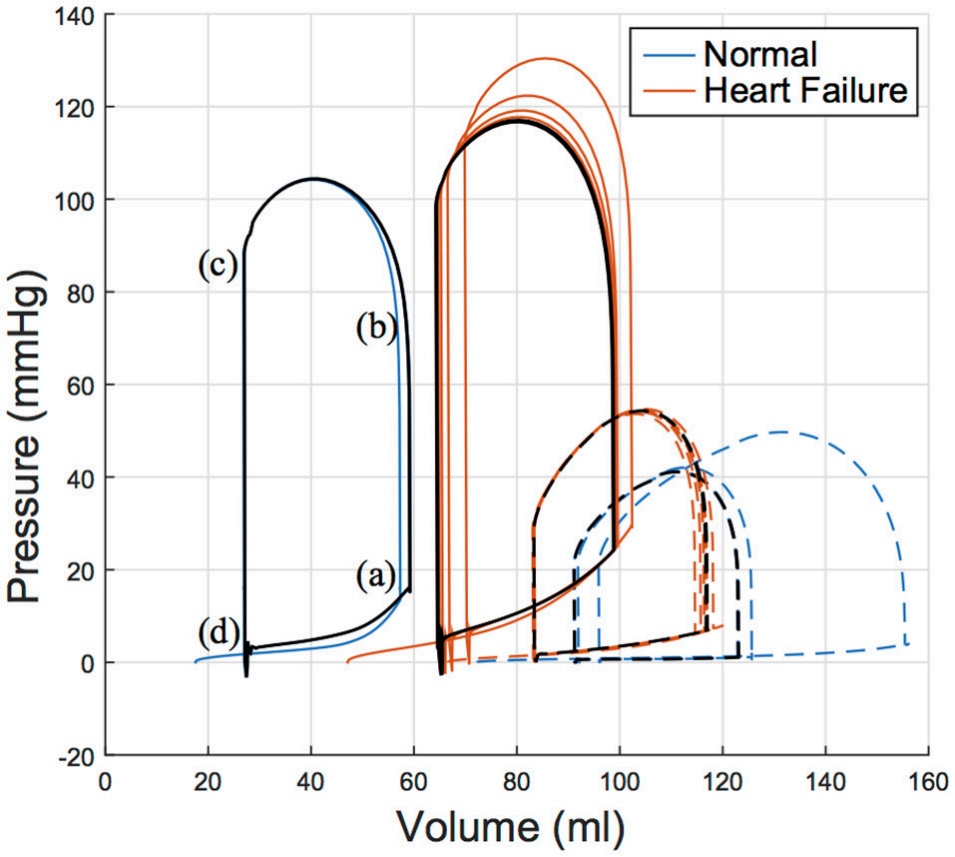
Pressure volume relation for the left ventricle (solid lines) and right ventricle (dashed lines) of both subjects over six simulated cardiac cycles with a heart rate of 77 bpm. The 5 and 6th cardiac cycle is plotted in black illustrating convergence. Key parts of the cardiac cycle are labeled on the LV PV loop for the normal (healthy) subject, which correspond to **(a)** end diastole, **(b)** start ejection, **(c)** end systole, and **(d)** end relaxation. LV, left ventricle; PV, pressure volume.

Cardiac function was simulated for six consecutive cardiac cycles (Figure 8) to ensure converged solutions were achieved. All values and results reported in the following sections correspond to the final and converged solution; i.e., these values will differ slightly from the calibrated targets that were acquired for only the first beat simulated, which is visible in Figure 8.

LV functional outputs compared reasonably between *in vivo* and *in silico* results. This is seen in SV, which was 32.2 vs. 30.9 ml in the normal subject for *in silico* and *in vivo* respectively, and 34.5 vs. 33.0 ml in the HF subject for *in silico* and *in vivo* respectively. Similarly, ejection fraction (EF) was 54.4 vs. 53.4% in the normal subject for *in silico* and *in vivo* respectively, and 35.0 vs. 32.0% in the HF subject for *in silico* and *in vivo*, respectively. These differences in SV and EF are considered minor when compared to the biological variations that occur from beat to beat.

Using the key parts of the cardiac cycle as presented in Figure 8, we assessed myofiber stress and strain values for each ventricle. These values were volumetrically averaged (i.e., normalized by element volume) to remove potential mesh artifacts. The mean volumetrically averaged myofiber stress is at its lowest at the end of relaxation (ER); it increases during passive filling, reaching a peak passive stress at end diastole (ED) and then rapidly increases during the systolic phase of the heart. By the start of ejection (SE), the myofiber stress is higher (order of magnitude greater than passive stress), which enables the continued contraction of the heart through ejection. While the myofiber stresses are high at end systole (ES), they continuously decline from this point, reaching the lowest values at ER, when the cycle repeats.

**Figure 9:**
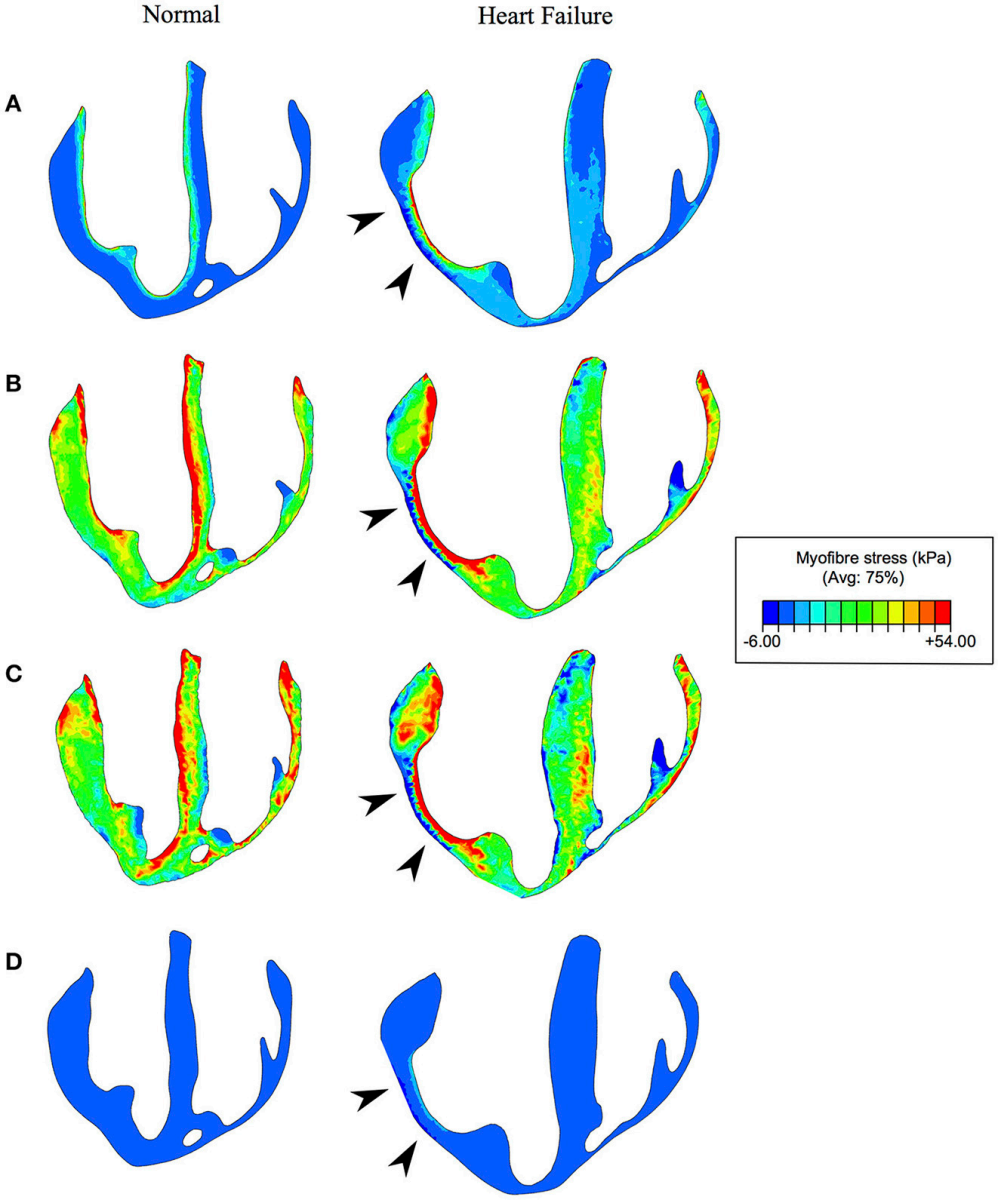
Myofiber stress at **(A)**: end-diastole, **(B)** start of ejection, **(C)** end-systole, and **(D)** end-relaxation. Left and right columns reveal the long axis cut planes of the ventricles for the normal (healthy) and diseased (heart failure) subjects respectively. The infarcted/fibrotic region is identified in the heart failure subject by black arrowheads. Non-symmetric contour limits were chosen to allow for a single set of limits to be used across the whole cardiac cycle.

The mean volumetrically-averaged myofiber stresses in the LV and RV are presented in Table 2. Additionally, myofiber stress contours are presented in Figure 9 over long-axis cut planes of the ventricular structure. These contour plots reveal qualitative details about the myofiber stress distributions associated with geometric position; e.g., peak stresses are seen on the endocardial surface of the LV.

**Table 2:** Volumetric-averaged mean myofiber stress results for the converged hearts presented separately for the LV and RV throughout the cardiac cycle.

	**LV myofiber stress (kPa)**		**RV myofiber stress (kPa)**	
**Time point**	**Normal**	**Heart failure**	**Normal**	**Heart failure**
ED	2.1 ± 4.2	4.7 ± 4.9	0.6 ± 0.6	3.5 ± 3.6
SE	23.4 ± 16.9	27.1 ± 18.9	21.1 ± 14	27.1 ± 16.9
ES	18.6 ± 14.9	24.4 ± 18.7	25.1 ± 18.3	24.8 ± 17.9
ER	0.0 ± 0.0	0.5 ± 0.5	0.1 ± 0.1	0.3 ± 0.4

*Results are presented with standard deviations. ED, end-diastole; SE, start-ejection; ES, end-systole; ER, end-relaxation*.

Since the HF subject had additional geometric information regarding the position and degree of pathological tissue (via the *h* field variable), we could further analyze stress by tissue health. Considering healthy tissue as regions whereby *h* = 1, infarcted tissue as *h* = 0, and border-zone tissue as values between these, we found mean myofiber stress was significantly different (*p* < 0.001) between all three regions, with substantially increased stress values within the infarcted tissue (Table 3).

**Table 3:** ED and ES volumetric-averaged mean myofiber stress results within the LV of the failing.

	**LV myofiber stress (kPa)**		
**Time point**	**Healthy tissue**	**Border-zone**	**Infarcted**
ED	4.1 ± 4.5	4.6 ± 4.9	10.5 ± 10
ES	23.2 ± 19.8	24.1 ± 21.1	39.4 ± 43.8

*Results are presented with standard deviations. ED, end-diastole; ES, end-systole*.

**Figure 10:**
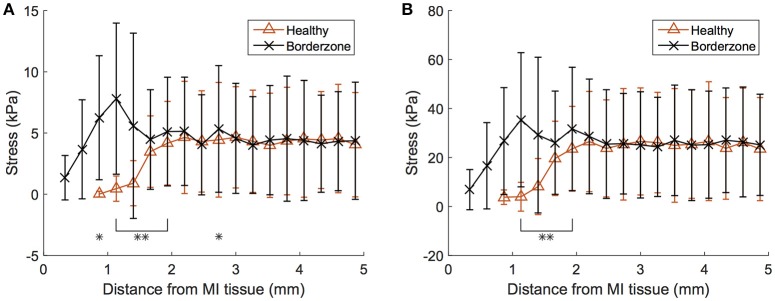
Myofiber LV stress within healthy and border-zone (i.e., 0 < *h* < 1) tissue presented by proximity to pathological tissue (within 5 mm from fully infarcted/fibrotic tissue). **(A)** Results shown for end-diastole and **(B)** end-systole for the failing subject. Error bars correspond to ± SD, ^*^p < 0.05, and ^*^^*^p < 0.01. MI, myocardial infarction.

The differences in stress values between normal and border-zone tissue are muted due to averaging. Analysis of these values as a function of geometric proximity to the fully infarcted/fibrotic tissue reveals more substantial differences (Figure 10). Most interestingly, the border-zone tissue experiences peak stresses roughly 1 mm away from the fibrotic tissue, after which they decrease and converge with healthy tissue values (distances >2 mm) for both ED and ES. Overlapping data points were analyzed for statistical significance and are illustrated in (Figure 10).

Similar to the analysis of myofiber stress, strain results were analyzed at the same key points in the cardiac cycle. The mean volumetrically averaged myofiber strains in the LV and RV are presented in Table 4. A detailed analysis of regional strain in the LV with respect to the local longitudinal and circumferential directions is presented in Section 3.5.

**Table 4:** ED and ES volumetric-averaged mean myofiber strain results for the converged hearts presented separately for the LV and RV.

	**LV myofiber strain (%)**		**RV myofiber strain (%)**	
**Time point**	**Normal**	**Heart failure**	**Normal**	**Heart failure**
ED	9.6 ± 6.7	6.9 ± 4.3	8.3 ± 5.7	5.5 ± 5.0
SE	−1.0 ± 10.4	1.0 ± 7.4	−4.3 ± 10	−2.6 ± 7.4
ES	−9.8 ± 5.0	−5.4 ± 6.5	−8.9 ± 6.5	−8.4 ± 3.7
ER	0.7 ± 2.0	1.9 ± 1.6	1.4 ± 2.4	1.0 ± 1.8

*Results are presented with standard deviations. ED, end-diastole; SE, start-ejection; ES, end-systole; ER, end-relaxation; LV, left ventricle; RV, right ventricle*.

### 3.5. Model validation

Initial comparison of the endocardial strains revealed strong agreement of global strains (i.e., averaged over the entire surface). For the normal subject, the global circumferential strain (GCS) for the FE simulations was −22.0%, which compares very well with the *in vivo* measurement of −21.9% from TomTec data. Global longitudinal strain (GLS) was −10.3% for the FE model simulation and −14.7% for the *in vivo* measurement. The HF subject produced similar comparisons: GCS for the FE simulations and the *in vivo* measurement were −14.4 and −12.7%, respectively, and GLS for the FE simulations and the *in vivo* measurement were −4.6 and −8.3%, respectively. In both subjects, circumferential strains were in closer agreement than longitudinal strains, which were both 4% lower than *in vivo* measurements from echocardiograms.

**Figure 11:**
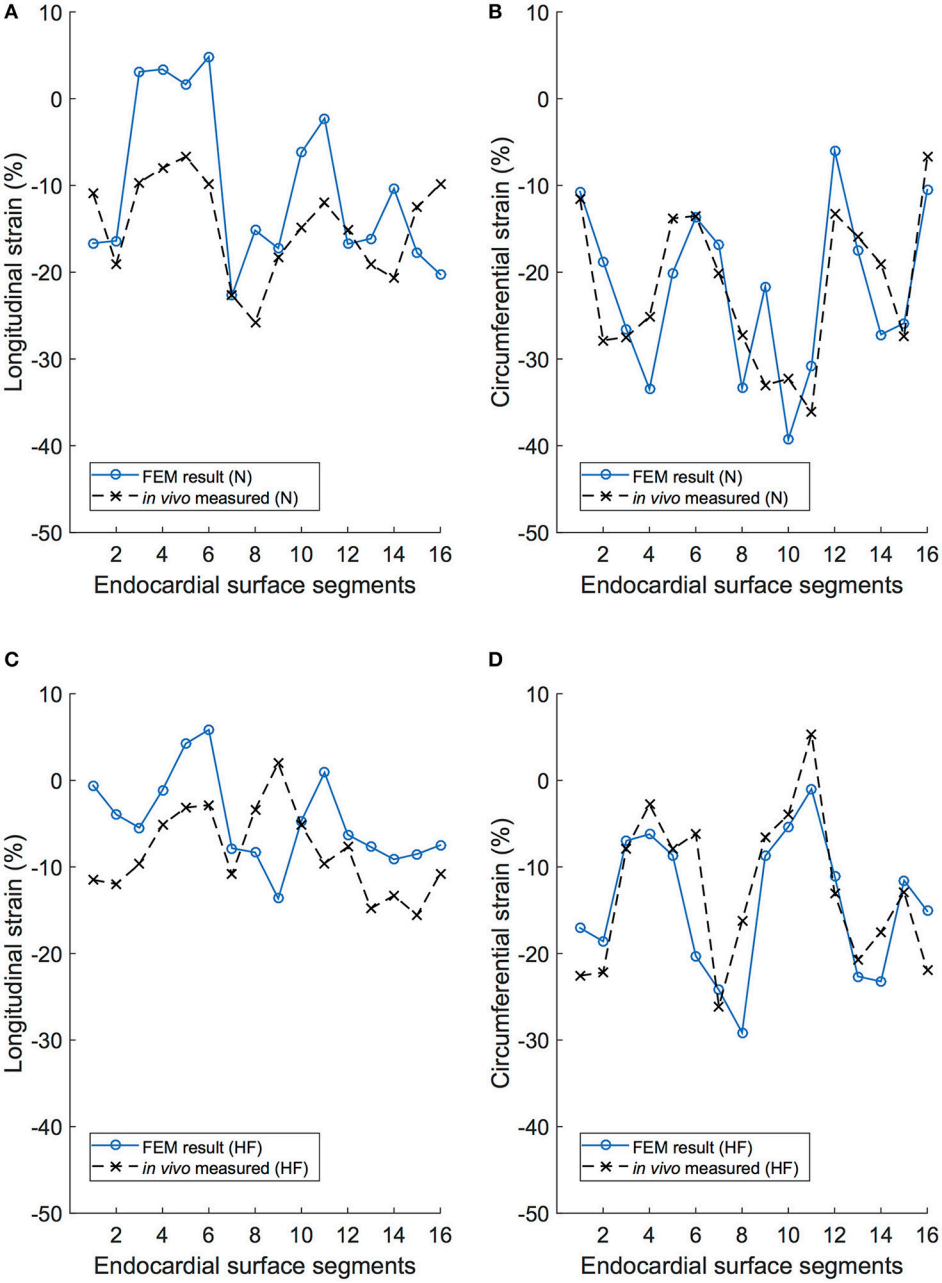
Longitudinal and circumferential endocardial strain comparison between the FE model (FEM) simulation and the *in vivo* recordings of the same porcine subject. **(A)** Longitudinal strain results for each of the 16 endocardial surface regions in the normal subject (N). **(B)** Circumferential strain results for each of the 16 endocardial surface regions in the normal subject (N). **(C)** Longitudinal strain results for each of the 16 endocardial surface regions in the heart failure subject (HF). **(D)** Circumferential strain results for each of the 16 endocardial surface regions in the heart failure subject (HF).

A regional analysis of comparison for FE model results and the *in vivo* measured values for strain on the endocardium surface reveals a very strong agreement in circumferential strains for both subjects, as seen in Figures 11B,D. Qualitatively, it is clear that the longitudinal strain behavior from the FE model correlates to the recorded *in vivo* values, with similar regional patterns displayed in both modalities, as seen in Figures 11A,C.

## 4. Discussion

The high degree of subject-specificity incorporated into these models vastly reduces the number of model assumptions needed to produce computational cardiac simulations. This has led to realistic mechanical behavior shown in the reported pressure-volume loops, our realistic determination of active tension (i.e., minimal cross-fiber contraction) and the independently reached strain behavior, which matches the measured strains from *in vivo* echocardiography.

### 4.1. Geometric segmentation

The geometric model construction used in this study is one of the most sophisticated ventricular structures produced for FE modeling of the heart compared to those found in the literature. While biventricular representations of the heart have become popular geometric choices for FE studies in recent years, most of these models truncate the geometry or exclude the papillary structures. The cardiac model from the Dassault Systèmes Living Heart Project (Baillargeon et al., 2014, 2015; Genet et al., 2016; Sack et al., 2016b) is an exception that does include this level of detail (and atrial structures); however, this heart geometry is not entirely patient-specific.

The geometric model of HF used in this study is a novel extension of how infarcted tissue has typically been presented in computational studies. Previously, studies have represented infarcted tissue using discrete concentric zones accounting for infarction and the border-zone as neat self-contained geometric regions (Wenk et al., 2011; Miller et al., 2013; Berberoglu et al., 2014). Our study incorporates subject-specific pathological detail, allowing for the description of infarcted material that ranges from concentrated to diffuse descriptions, as can be seen in Figure 2. Moreover, this level of subject-specific geometric detail is coupled with subject-specific fiber detail accounting for regionally precise myofiber detail throughout the ventricular structure, which has never been included in models with HF.

### 4.2. Subject-specific myofiber orientation

The critical role of myofiber orientation on mechanical and electrical function is well recognized (Beyar and Sideman, 1984b; Bovendeerd et al., 1992; Chen et al., 2005; Bishop et al., 2009; Wang et al., 2013). Fiber orientation, along with a second orthogonal vector within the laminar sheet, provides sufficient information to establish a local orthonormal coordinate system whereby each basis vector corresponds to a principal anisotropic direction. To the best of our knowledge, the present models are the first mechanical models to include subject-specific local coordinates derived from DTMRI.

The values of α_h_ in the normal subject conform well to other studies of large animals in terms of mean values (Streeter and Bassett, 1966; Streeter et al., 1969; Nielsen et al., 1991; Geerts et al., 2002; Helm et al., 2005; Ennis et al., 2008) and typical standard deviations expected from regional DTMRI myofiber analysis (Scollan et al., 1998; Lombaert et al., 2012). Our data reveals larger angles (i.e., close alignment with the longitudinal direction) in the anterior and inferior endocardial region when compared to other AHA regions circumferentially. These regions typically contain large papillary muscles on the endocardium, which explain this observation. These endocardial values of α_h_ in the anterior and inferior regions tend to plateau with values before declining linearly as a position of wall depth. The study of fiber orientation in papillary muscles is relatively unreported in the literature, due to difficulty in segmentation from *in vivo* imaging. In the early experimental work of Streeter et al. (1969), however, the authors also identified a plateau of myofiber angle α_h_ of roughly 90° in papillary muscles.

Myofiber orientations in the failing heart have striking similarities to the healthy heart in anterior, inferior, and the majority of septal (i.e., excluding basal anteroseptal) regions. Our results show that the largest discrepancies in myofiber angles correspond to regions in the LV free wall (i.e., basal and mid lateral regions), regardless of underlying fibrotic tissue content. This suggests that together with underlying pathology, mechanical factors relating to the position and function of an LV region are linked to its susceptibility to remodel.

Lower values of the myofiber inclination angle α_h_ in the vulnerable anterolateral regions are consistent with values reported in other studies investigating change in fiber orientation due to infarction (Holmes et al., 1997; Wu et al., 2007, 2009). In contrast, the inferolateral regions in the current HF model present with higher values of α_h_, especially near the endocardial surface. Here, α_h_ plateau with values highly aligned longitudinally, indicative that the inferior papillary muscle was segmented within these regions and may be clouding the results. Even with consistent segmentation techniques, biological variance between subjects is an unavoidable challenge when sources of discrepancies are being determined. This likely explains the disagreement seen in regions with little fibrotic content.

### 4.3. Passive material estimation

The choice to use human shear experimental data, instead of porcine data from an earlier study (Dokos et al., 2002) was made after we calibrated our model to both and found that the porcine data produced *much* stiffer material behavior, especially in the fiber direction. As the human study was performed over a decade after the porcine study, more sophisticated methods were utilized in preventing contracture of heart muscles in the specimen extraction process, making the results likely more reliable representations of *in vivo* material response. Our calibration methods are proficient in capturing experimentally recorded orthotropic material response, as seen in Supplementary Figure S2, and when scaled, match the predicted Klotz curve for passive filling accurately (Figure 7). This two-stage method of cardiac tissue calibration, which utilizes small specimen data and pressure-volume data, is becoming a common method for obtaining realistic (and subject-specific) parameters (Krishnamurthy et al., 2013; Gao et al., 2015; Sack et al., 2016c). It ensures that the resulting material parameters produce a material that conforms to the anisotropy uncovered from mechanical experimentation and realistic *in vivo* function.

The determination of passive material parameters for the myocardial tissue ultimately depends on three factors: (1) the unloaded volumes V_0_ of the ventricles; (2) the target ED volumes; and (3) the assumption regarding infarct stiffness. Our calibration techniques found that the HF subject yielded material parameters roughly an order of magnitude greater (considering the linear scaling coefficient A) (Table 1) than the normal subject, resulting in a much stiffer passive filling curve (Figure 7). This increase in remote stiffness is in line with other studies that have found that the remote tissue experiences changes in material properties (Bogaert et al., 2000; McGarvey et al., 2015), as well as the underlying physiological process of adverse remodeling, which results in collagen content, deposition, and cross-linking increasing in both infarcted and remote regions of the heart (Holmes et al., 2005; van den Borne et al., 2008; Fomovsky and Holmes, 2010). Another noteworthy finding is that the unloaded LV cavity in the subject with HF is almost three times as large as that of the healthy counterpart (Table 1).

### 4.4. Active material estimation

Contraction is initiated by sarcomere shortening in series, which in turn contracts myofibrils. Whereas the mechanical analysis of this multi-scale phenomenon would seem to only occur in the myofiber direction, contraction has also been recorded in cross-fiber directions (Lin and Yin, 1998), which has been linked to the splay and dispersion of myofibers. The active material calibration resulted in *T*_*max*_ = 114.0 kPa and *n*_*s*_ = 0.07 in the normal subject, and *T*_*max*_ = 140.6 kPa and *n*_*s*_ = 0.14 in the HF subject. The value found for *T*_*max*_ is in line with the values reported in Genet et al. (2014): 130–155 kPa. These values of *n*_*s*_ are typically lower than those found in cardiac models that use generic fiber descriptions, which typically apply 40% of active stress in this direction (i.e. *n*_*s*_ = 0.4) (Lee et al., 2013; Genet et al., 2014; Zhang et al., 2015). Our lower values of *n*_*s*_ are due to the accurate subject-specific fiber orientations incorporated in the model, which naturally reduces the inclusion of unrealistically high (and potentially non-physiological) cross-fiber contraction.

### 4.5. *In silico* subject-specific heart

The patient-specific metrics used to calibrate the material model of the heart are preserved in the converged cardiac-cycle simulation with an error <5%. This is hardly a shortcoming as a physiological *in vivo* heart operates within a range of values, often experiencing variances in SV, pressure and timing from cardiac cycle to cardiac cycle. Without any subject-specific pressure-volume data for the RV, we assumed the same material properties as the LV and loaded the RV using values derived from the literature. While it appears this initial loading state was far from the converged solution, it had comparatively little impact on the LV function. Rather, the RV experienced the majority of the functional change to ensure hemodynamic equilibrium. It is reassuring to see that despite the lack of appropriate data for the RV, the combined ventricular function converges to a state closely in line with LV *in vivo* targets. This unidirectional ventricular dependence is pronounced in the porcine heart, where the LV is overwhelming the major mechanical component of the heart, and may be less pronounced in human models.

Qualitatively, the strain results conform to expectations. The myofiber elongation is at its greatest at ED, is at its minimum during systole (due to the contractile material behavior), and returns to almost the original length before passive filling starts (Table 4). Furthermore, these strain results reveal functional changes due to pathology. The failing heart experiences diminished myofiber strains within the LV at ED (6.9 ± 4.3 vs. 9.6 ± 6.7) due to its stiffer material composition, and diminished myofiber strains at ES (−5.4 ± 6.5 vs. −9.8 ± 5.0) due to the loss of contractile function in the infarcted region.

An accurate determination of stress within complex mechanical problems is an unrivaled advantage of computational modeling. This is especially relevant for cardiac mechanics as changes in ventricular wall stress are thought to initiate pathological remodeling (Pfeffer and Braunwald, 1990; Sutton and Sharpe, 2000; Matiwala and Margulies, 2004). A unique problem pertaining to biological materials is that *in vivo* stresses cannot be accurately replicated under *in vitro* experimental protocols and thus, cannot be measured directly (Dorri et al., 2006). Our results show that chronic HF results in increased mean myofiber stresses in both ventricles at ED, and in the LV at ES (Table 2). This is interesting in the context of our strain results; i.e., the subject with HF experiences increased stress while simultaneously experiencing reduced strain. Determining stress directly from strain, without accounting for the anatomic features of the infarcted tissue or a proper constitutive characterization of the myocardial tissue (e.g., in Laplace's law), could lead to highly erroneous conclusions about the actual stress state within the failing heart. This has been demonstrated by Zhang et al. (2011), who compared Laplace's law and FE methods to evaluate stress in an infarcted LV. Their analysis showed that the average stress from Laplace's law is significantly different to the comprehensive stress analysis produced through anatomically accurate FE techniques.

While these increases in myofiber stress are prevalent throughout the myocardium, the infarcted region experiences myofiber stress roughly twice as high as in remote regions (Table 4). Furthermore, the myocardial wall containing the infarcted region, which is geometrically thinner than other regions, experiences complex stress due to its morphology and stiffer, non-contractile material behavior. Along the endocardium it experiences extremely high tensile stresses and along the epicardium it experiences compression (Figures 9A–C). We also discovered that in border-zone regions peak stresses occurred roughly 1 mm from fully infarcted tissue. Since stress is thought to initiate pathological remodeling (Pfeffer and Braunwald, 1990; Sutton and Sharpe, 2000; Matiwala and Margulies, 2004), this may explain the mechanical propagation of infarct expansion.

Due to the lack of comparable porcine computational studies we compared our stress results to available human studies. We found that the LV myofiber stresses in the normal subject compare well with literature values; e.g., our predicted ED stress of 2.1 ± 4.2 kPa vs. 1.47 ± 20.72 (Sack et al., 2016b) and 2.21 ± 0.58 (Genet et al., 2014).

### 4.6. Model validation

The comparison between *in vivo* and model-predicted strains resulted in very similar qualitatively and quantitative results. For global values, the circumferential strains matched very well (<0.1% error), whereas the longitudinal strains were under-predicted in the FE model by merely 4%. A regional analysis reinforced this–circumferential strains matched extremely well for both subjects (Figure 11) and longitudinal strains matched with less accuracy (particularly in HF). The mismatch between longitudinal strain results may be due to simplifications inherent in the derivations of strains from echocardiography or our FE model. One the one hand, the surface resolution of echocardiography is typically low resolution (likely excluding finer morphological features), whereas the FE model has a much higher endocardial surface resolution. Echo-derived strains exclude the papillary muscles, and are often determined “sub-endocardially.” These papillary muscles, which pull downward during systole, would clearly alter the longitudinal strain measurement when included in the determination. On the other hand, our FE model excludes the atria and the pericardium. Ventricular motion is likely coupled with the presence (and weight) of the atria and their function (Fritz et al., 2014), which would specifically affect longitudinal strains. To further complicate the comparison, the difficulty in accurately measuring *in vivo* strains from echocardiography is well documented. For example, consistent strain-data reproducibility (intra- and inter-observer) is the subject of multiple medical studies, some showing very poor outcomes (Gayat et al., 2011; Badano et al., 2012). Currently, the reliability of global deformation metrics from echo-derived 3D strain is strong, (i.e., GCS and GLS), but the reliability to accurately quantify regional deformation is not (Lang et al., 2015). The uncertainty regarding the reliability of regional strains (from either modality) requires further study either using full-heart models or a better source of *in vivo* strain data (i.e. MRI-derived).

### 4.7. Clinical translation

Reliable computational heart models offer the potential to integrate diverse data, produce otherwise unobtainable metrics relating to function, and quantify complex coupled mechanical behavior in an unparalleled manner. This can be especially advantageous as a research modality investigating the efficacy of treatments pre-clinically. Computational models allow for therapeutic parameters to easily be perturbed, whereby various *in silico* experiments can be investigated to determine optimal treatment efficacy.

As computing resources become cheaper and more efficient, computational modeling is increasingly being viewed as a viable complementing modality in the clinic. As shown in this study, reliable subject-specific models are achievable with the proper inclusion of high quality imaging data. As imaging technology becomes more advanced, it will become feasible to replicate the quality of models using purely *in vivo* imaging data. This would enable the ability to investigate potential therapies *in silico* prior to their application to patients–with medical decisions, treatment plans and interventions being highly patient-specific.

Additional techniques are needed to integrate clinical data into the methods presented here. Firstly, methods that can indirectly assess the unloaded geometry from an *in vivo* representation, such as inverse-displacement techniques (Bols et al., 2013; Rausch et al., 2017), will likely be required. Secondly, if pressure catheterization data is not available, methods to approximate or infer patient pressures will be needed. Cuff pressures could be used to identify the pressure range in the systemic arteries and Nagueh's formula (Nagueh et al., 1997), can be employed on echocardiogram data to obtain approximate LV filling pressures.

### 4.8. Model limitations

While geometrically detailed, our computational model is still lacking physiological features such as the atria, the pericardial sac and the diaphragm. This may be the source of discrepancies seen in longitudinal strains, especially among basal regions. For the normal subject, an average value of EDP had to be used when determining the *in vivo* target. A further shortcoming of this work is that it has only been applied to a single subject for each condition, but our current research is focused on expanding this to a larger cohort and including failing hearts treated with biomaterial injection therapy. Our material model of the heart is limited in that it does not include dispersion, micro-structural mechanics or regional heterogeneity of the tissue. Finally, only the mechanical component of heart function was simulated and excluded electro-chemical-mechanical considerations (i.e., the polarization of tissue, excitation and propagation phenomena).

## 5. Conclusions

This study introduces subject-specific cardiac models for biventricular porcine heart models in healthy and diseased states. Subject specificity is introduced through geometric features, local myofiber directions, loading conditions, hemodynamics and the distribution of fibrotic tissue in the failing heart. These models were calibrated to *in vivo* subject-specific metrics, and were able to accurately capture functional outputs such as SV and EF. The close global and regional agreement between *in vivo* and *in* silico strains illustrated the success of our methods to create computational models that can serve as *in silico* surrogates for real hearts in healthy and diseased states. This level of agreement, and therefore validation, is a milestone for cardiac computational modeling. As such, stress and strain values presented in this study (for both ventricles and at multiple time points during the cardiac cycle) can serve as a guideline for future studies.

## Author contributions

KS, JG, and TF were involved in the conception and design of study. KS created the computational models. Animal experiments were performed by JC and GK. Acquisition of various data critical to model creation was performed by JC, EA, and DE. The analysis and interpretation of modeling results was performed by all authors. KS wrote the first draft of the manuscript. All authors contributed to manuscript revision, read and approved the submitted version.

### Conflict of interest statement

The authors declare that the research was conducted in the absence of any commercial or financial relationships that could be construed as a potential conflict of interest.
