# Supplementary material for: Construction and Validation of Subject-Specific Biventricular Finite-Element Models of Healthy and Failing Swine Hearts From High-Resolution DT-MRI
Source: Front Physiol. 2018 May 29;9:539. doi: 10.3389/fphys.2018.00539 (PMC5986944; doi:10.3389/fphys.2018.00539)
Supplement: Supplementary file 1 [file Data_Sheet_1.pdf]

Supplementary Material

Construction and validation of subject-specific biventricular finite-element models of healthy and failing swine hearts from high-resolution DT-MRI

Kevin L. Sack[1, 2], Eric Aliotta[3], Daniel B. Ennis[3], Jenny S. Choy[4], Ghassan S. Kassab[4], Julius M. Guccione[2*], Thomas Franz[1, 5]

[1] Division of Biomedical Engineering, Department of Human Biology, University of Cape Town, Cape Town, South Africa

[2] Department of Surgery, University of California at San Francisco, San Francisco, California, USA

[3] Department of Radiological Sciences, University of California, Los Angeles, California, USA

[4] California Medical Innovations Institute, Inc., San Diego, California, USA

[5] Bioengineering Science Research Group, Engineering Sciences, Faculty of Engineering and the Environment, University of Southampton, Southampton, UK

*** Correspondence:**
Julius M Guccione
Email: Julius.Guccione@ucsf.edu
Phone: 415 680 6285
Fax: 415 750 2181

1 Supplementary Figures and Tables

Table S1: In vivo volume and pressure target values used in the model creation. Abbreviations: EDV: End diastolic volume; SV: Stroke volume; EDP: End diastolic pressure; ESP: End systolic pressure. * Mean value from full data set of larger group (n = 5) at healthy baseline.

Measurement	Healthy Subject	HF Subject	Source
LV EDV (ml)	57.8	103.0	*in vivo* echocardiogram
LV SV (ml)	30.9	33.0	*in vivo* echocardiogram
LV EDP (mmHg)	13.5 *	29.6	*in vivo* pressure catheterization
LV ESP (mmHg)	87.6 *	130	*in vivo* pressure catheterization
Min aortic pressure	60.6 *	90	*in vivo* pressure catheterization

Table S2: Constitutive parameters for the passive material response.

Passive Parameters	Description
a, b	Governs the isotropic response of the tissue
a_f, b_f	Governs additional stiffness in the fibre direction
a_s, b_s	Governs additional stiffness in the sheet direction
a_{fs}, b_{fs}	Governs coupling stiffness in the fibre and sheet directions
I_1	The first isochoric strain invariant
I_{4i}	A pseudo-invariant defined as $\boldsymbol{A}_i \cdot \widetilde{\boldsymbol{C}} \cdot \boldsymbol{A}_i$
I_{8fs}	A pseudo-invariant defined as $\boldsymbol{A}_f \cdot \widetilde{\boldsymbol{C}} \cdot \boldsymbol{A}_s$
$\widetilde{\boldsymbol{C}}$	The isochoric Right Cauchy-Green tensor ($\widetilde{\boldsymbol{C}} = J^{-2/3}\boldsymbol{C}$)
$\boldsymbol{A}_i$	Vector in direction i
D	Multiple of Bulk modulus ($K = 2/D$)
J	The third deformation gradient invariant ($J = \det \boldsymbol{F}$)
**Active Parameters**	**Description**
t_0	Time to reach peak tension after the initiation of active tension
m	Governs the slope of the relaxation
b	Governs the length of relaxation
l_0	The sarcomere length below which no active force develops
B	Governs the shape of the peak isometric tension-sarcomere length relation
Ca_0	The peak intercellular calcium concentration
$Ca_{0_{max}}$	The maximum intercellular calcium concentration
T_{max}	The maximum active tension able to develop
l_r	The initial sarcomere length
E_{ff}	Green strain in the fiber direction

Table S3: Control values for the lumped circulatory flow model and literature values for comparison.

Variable	Units	Both subjects	Literature
R_M	mmHg s ml^{-1}	0.03	0.0 - 0.20 [1-3]
R_A	mmHg s ml^{-1}	0.03	0.0 - 0.20 [1-3]
R_{SYS}	mmHg s ml^{-1}	1.70	1.05 - 4.00 [1-3]
R_P	mmHg s ml^{-1}	0.03	0.0 - 0.06 [1-3]
R_T	mmHg s ml^{-1}	0.03	0.0 - 0.02 [1-3]
C_P	ml mmHg^{-1}	4.43	1.0 - 7.0 [2-4]
C_{SA}	ml mmHg^{-1}	0.80	0.3 – 2.1 [2-4]
C_{SV}	ml mmHg^{-1}	16.6	6.0 - 17.0 [2, 3]

Table S4: Initial pressure state of the compliance vessels in the subject-specific model.

Cavity	Units	Healthy subject	HF subject	Motivation
C_P	mmHg	15	35	Needed to ensure LV fills to correct pressure
C_{SA}	mmHg	65	90	Needed to ensure ejection begins at correct pressure
C_{SV}	mmHg	4	8	Needed to ensure RV fills to correct pressure
LV	mmHg	13.5	29.6	Determined from *in vivo* data
RV	mmHg	4	8	Based on literature: 1-7mmHg [5] 3.9 ±1.6 [6]

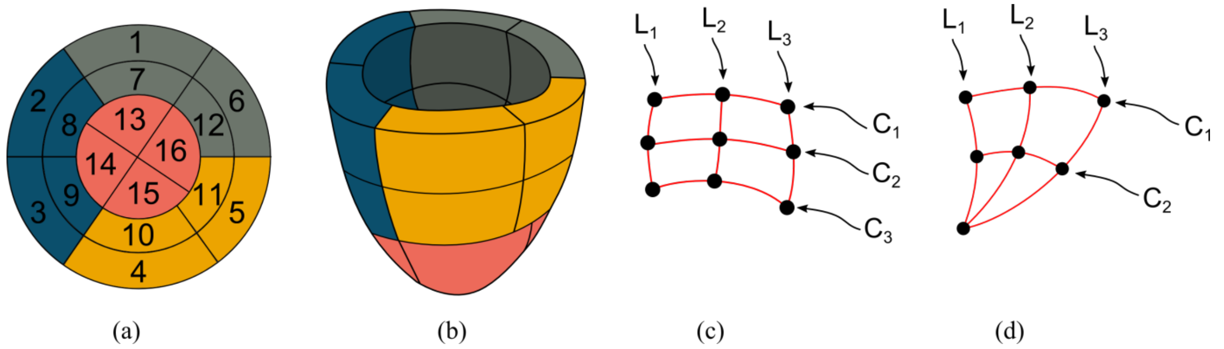

Figure S1: The 16 LV segment breakdown used in the in vivo strain calculation illustrated on (a): a radial map and (b): a simplified LV model. (c): A quadrilateral surface segment defined by 9 nodes. Nodes are joined by 3 circumferential cubic splines, C_i, and 3 longitudinal cubic splines, L_i. (d): A triangular surface segment defined by 7 nodes. Nodes are joined by 2 circumferential cubic splines, C_i, and 3 longitudinal cubic splines, L_i.

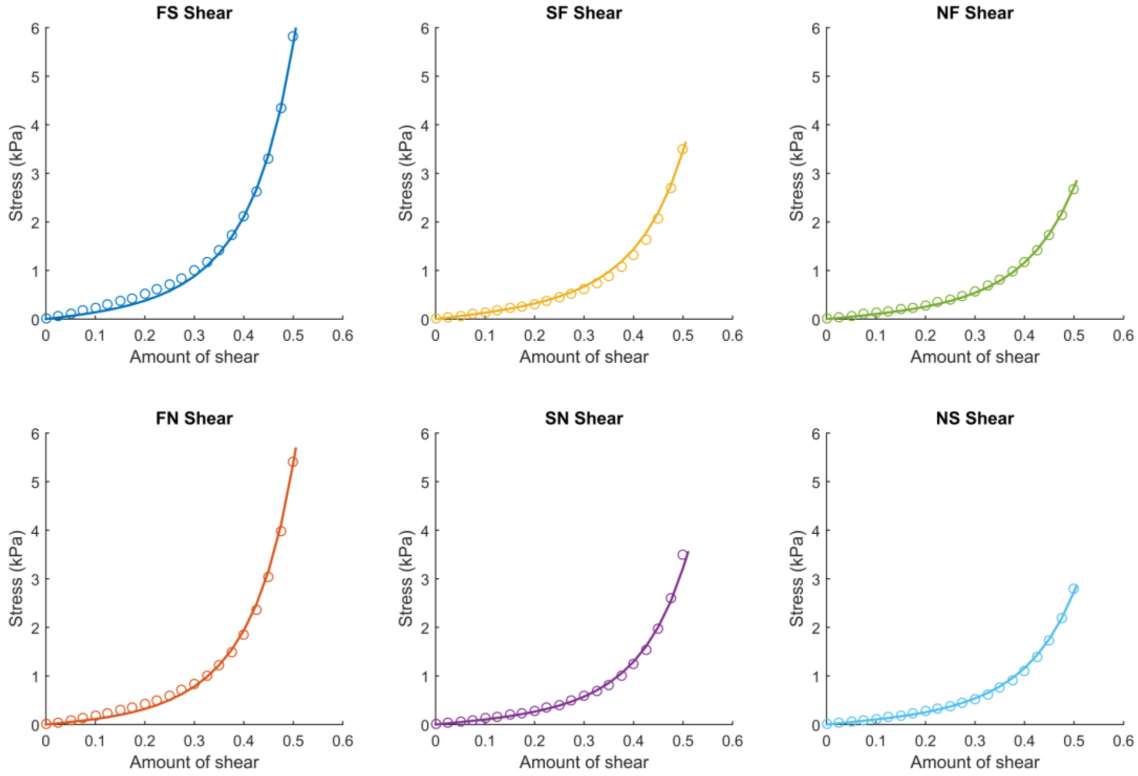

Figure S2: Passive material response of all 6 shear modes of an idealized tissue cube. FE model response given by solid lines and experimental data presented as circles.

2 Additional details for active contraction

The full description of active tension is described by

$$T_a(t,l) = T_{MAX} \frac{Ca_0^2}{Ca_0^2 + ECa_{50}^2(l)} \frac{\left[1 - \cos\left(\omega(\text{mod}(t), l)\right)\right]}{2} h \tag{S1}$$

where

$$ECa_{50}(l) = \frac{Ca_{0_{max}}}{\sqrt{e^{B(l-l_0)} - 1}}, \tag{S2}$$

Due to the cyclic nature of the cardiac cycle, the modulus function acting on time is enforced to wrap the time variable around after each cardiac. This enforces $0 \leq \text{mod}(t) \leq HR/60$. Where HR is the heart rate. In our case HR is chosen as 77 Bpm for both subjects.

$$\omega(\text{mod}(t), l) = \begin{cases} \pi \dfrac{\text{mod}(t)}{t_0}, & \text{when } 0 \leq \text{mod}(t) \leq t_0 \\ \pi \dfrac{\text{mod}(t) - t_0 + t_r(l)}{t_r}, & \text{when } t_0 \leq \text{mod}(t) \leq t_0 + t_r(l) \\ 0, & \text{when } \text{mod}(t) \geq t_0 + t_r(l) \end{cases} \tag{S3}$$

$$t_r(l) = ml + b, \tag{S4}$$

$$l = l_r\sqrt{2E_{ff} + 1}, \tag{S5}$$

with parameters definitions provided in Supplementary Table1 and baseline values taken from [7]. This mathematical description of active tension ensures a smooth yet steep transition from zero tension at the start of systole to peak active tension, T_{MAX}, at time t_0 and then a smooth decline back to zero for the specified relaxation time t_r.

Supplementary references

1. Hoppensteadt, F.C. and C.S. Peskin, *Mathematics in medicine and the life sciences*. 1992, New York, NY: Springer-Verlag.
2. Pilla, J.J., J.H. Gorman III, and R.C. Gorman, *Theoretic Impact of Infarct Compliance on Left Ventricular Function.* Annals of Thoracic Surgery, 2009. **87**(3): p. 803-810.
3. Santamore, W.P. and D. Burkhoff, *Hemodynamic consequences of ventricular interaction as assessed by model analysis.* American Journal of Physiology-Heart and Circulatory Physiology, 1991. **260**(1): p. H146-H157.
4. Watanabe, H., et al., *Multiphysics simulation of left ventricular filling dynamics using fluid-structure interaction finite element method.* Biophysical journal, 2004. **87**(3): p. 2074-2085.

5. Mann, D.L., et al., *Braunwald's heart disease*. 2015, Philidelphia, PA: Elsevier-Saunders.

6. Quinn, T.A., et al., *Effects of sequential biventricular pacing during acute right ventricular pressure overload.* American Journal of Physiology-Heart and Circulatory Physiology, 2006. **60**(5): p. H2380.

7. Walker, J.C., et al., *MRI-based finite-element analysis of left ventricular aneurysm.* American Journal of Physiology-Heart and Circulatory Physiology, 2005. **289**(2): p. H692-H700.
